# Clinical presentation of paediatric patients with COVID-19 admitted to a single paediatric intensive care unit (PICU) in Iran

**DOI:** 10.1136/bmjpo-2020-000715

**Published:** 2020-09-17

**Authors:** Amir Saeed, Eslam Shorafa, Anahita Sanaeidashti, Mohammad Rahim Kadivar

**Affiliations:** 1 Department of Pediatrics, Shiraz University of Medical Sciences, Shiraz, Iran; 2 department of pediatrics, division of intensive care unit, Shiraz University of Medical Sciences, Shiraz, Iran

**Keywords:** health services research, virology, mortality

## Abstract

**Objectives:**

To describe the clinical characteristics of paediatric patients admitted to a single paediatric intensive care unit (PICU) in Iran with COVID-19.

**Methods:**

A cross-sectional study of paediatric patients who were admitted to a COVID-19-dedicated PICU from 16 March 2020 to 21 April 2020 with COVID-19.

**Results:**

Six children had confirmed COVID-19 and four had suspected COVID-19. Six had pre-existing chronic medical conditions. Nine had respiratory failure and needed ventilation. Five children, of whom four had chronic medical conditions, died. Four had cardiac arrhythmias. Clinical presentation included fever and cough.

**Conclusion:**

COVID-19 can be fatal in paediatric patients, especially in those with a chronic medical condition.

**What is known about the subject?**

- COVID-19 infection is less frequent and severe in paediatric patients than adult patients.
- The main symptom is fever and few paediatric patients need mechanical ventilation or inotrope support.

**What this study adds?**

- COVID-19 infection may be fatal in paediatric patients, especially in those with chronic medical conditions.
- The main respiratory problems in severe cases are oxygenation failure and a hypotensive state necessitating inotropes.
- The main causes of mortality in our study were cardiac arrhythmia and refractory hypoxemia.

## Introduction

The coronavirus pandemic originated in Wuhan, China^1 2^. The current outbreak of infections with SARS-CoV-2 was termed COVID-19 by the WHO.^3^ The disease rapidly spread from Wuhan to other areas of the world, so that the WHO announced that the outbreak was a pandemic in March 2020.^4^

The first paediatric case was a 10-year-old Chinese boy, whose family had visited Wuhan City.^4^A retrospective study on 366 children hospitalised for respiratory infections in January 2020 confirmed COVID-19 infection in 6 (1.6%) of them. This study suggests that COVID-19 infections in children occurred early in the epidemic.^5^ Children were rarely tested for the virus in the earlier phase of the outbreak, so there are limited data on the prevalence of COVID-19 in children.^6^ The virus can produce a Kawasaki-like illness in children. WHO developed a preliminary case definition for this condition which was later named ‘multisystem inflammatory disorder’ in COVID-19 (MIS-C).^7^

In Iran, the first COVID-19 cases were detected in February 2020 in Qom city. There are a few reports from critically ill paediatric patients in the country. In this article, we describe the characteristics of paediatric patients with COVID-19 admitted in paediatric intensive care unit (PICU) of Namazi Hospital in Shiraz, Iran.

## Methods

Namazi Hospital, in Shiraz, is the largest and the main tertiary referral centre in the south of Iran with more than 1000 beds. The medical PICU of this hospital has 18 beds with two separate sections, one of which was devoted to COVID-19 cases from the beginning of the outbreak and those with suspected or confirmed COVID-19 were admitted in this ward. All children aged 1 month to 18 years admitted to the COVID-19 PICU between 16 March and 21 April were included.

A confirmed case of COVID-19 was defined by a positive result on a reverse transcriptase PCR (RT-PCR) assay of a specimen collected on an oropharyngeal swab, nasopharyngeal swab or bronchoalveolar lavage. The tests were performed using an Applied Biosystem Step One plus real-time PCR machine (Applied Biosystem, California, USA). Amplification of N and ORF1b took place in a 20 µL single-tube and Superscript III Platinum one-step quantitative RT-PCR system (Invitrogen, Carlsbad, California, USA). Reactions contained 10.0 µL of 2X RT/PCR reaction mix, 1 µL primers/prob mix, 0.4 µL Superscript III RT/Platinum Taq mix, 0.4 µL ROX reference dye and 5 µL of extracted sample RNA or serially diluted previously confirmed patients’ positive control. The cycling conditions consisted of one cycle at 50°C for 10 min, one cycle at 95°C for 2 min, 45 cycles at 95°C for 5 s and 60°C for 30 min.^8^

**Figure 1:**
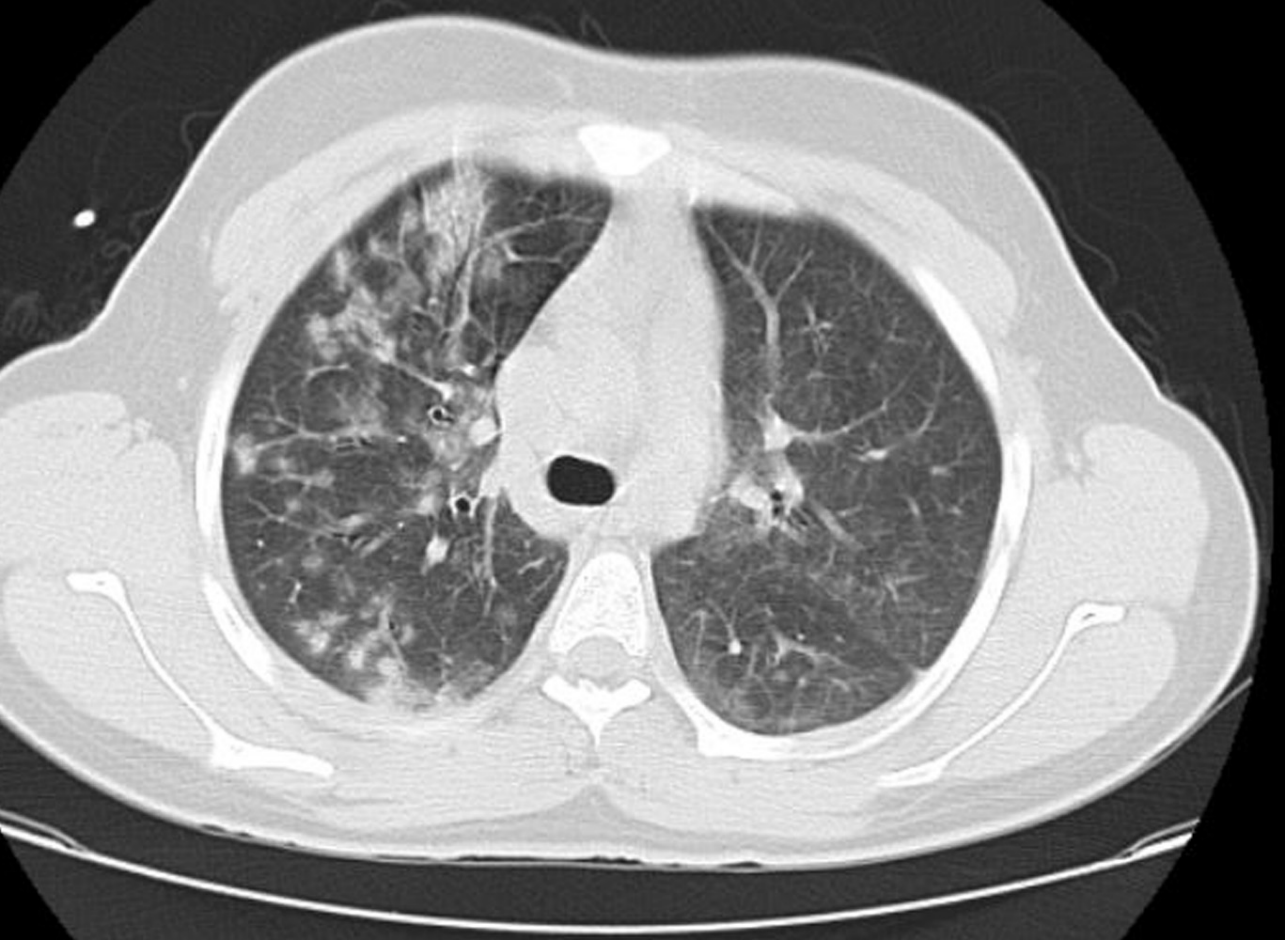
Chest CT of patient number 5.

**Figure 2:**
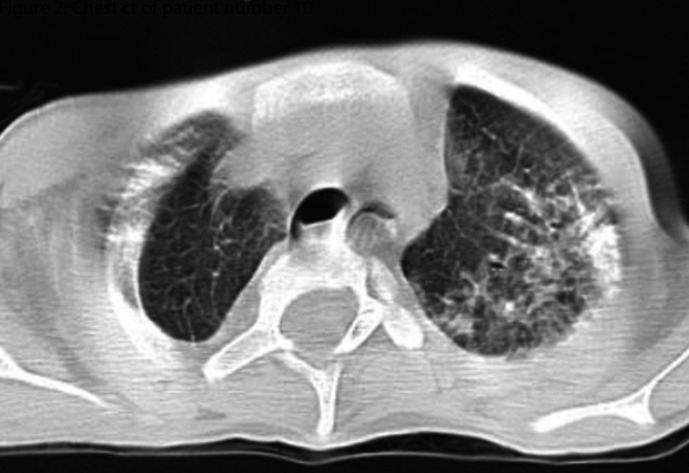
Chest CT of patient number 10.

A suspected case of COVID-19 was defined as a patient with clinical and laboratory findings^9^ (table 1) plus chest CT findings consistent with COVID-19 infection^10^ (figures 1 and 2) and history of close contact, but negative PCR result.

**Table 1:** Laboratory and clinical findings in favour of COVID-19 infection

Laboratory findings		Clinical manifestation
Increased	Decreased	
CRP D.dimer	Albumin lymphocytes	Fever, cough, sore throat, headache, shortness of breath, nausea, vomiting, abdominal pain
ESR ferritin		
AST, ALT procalcitonin		Sepsis and organ failure
LDH		

ALT, alanine aminotransferase; AST, aspartate aminotransferase; CRP, C-reactive protein; ESR, erythrocyte sedimentation rate; LDH, Lactate dehydrogenase.

## Results

From 16 March 2020 to 21 April 2020, six patients were admitted with confirmed COVID-19 and four had suspected COVID-19 (tables 2 and 3). Only one of them was transferred from the emergency department with suspected COVID-19. Nine were transferred from other wards with other suspected diagnoses. The median age was 7.9 years. Eight patients were men, six patients had chronic medical conditions (table 2). The other four were previously healthy.

**Table 2:** Clinical details of the patients

Patient number	1	2	3	4	5	6	7	8	9	10
Real-time PCR	+	+	+	+	+	+	–	–	–	–
Age(years)	4 months	3	11	13	15	16	1	1.5	3	16
Sex	Male	Male	Male	Male	Male	Male	Male	Female	Female	male
Hypotension	Yes	Yes	Yes	Yes	No	Yes	Yes	Yes	Yes	Yes
Tachycardia on admission	Yes	Yes	Yes	Yes	Yes	Yes	Yes	Yes	Yes	Yes
Tachypnoea on admission	Yes	Yes	Yes	Yes	Yes	Yes	Yes	Yes	Yes	Yes
Chief complaint(s)	Fever and letharginess	Fever and cough	Fever and abdominal pain and decreased LOC	Fever and letharginess	Fever and cough	Fever and cough	Fever and letharginess	Fever and cough	Fever and cough	Fever and cough
Previous illness	Cerebral Shunt	Negative	Negative	Cerebral palsy	Negative	Wegener granolomatosis	UPJO with nephrostomy	Negative	Immunodeficiency*	Cerebral palsy
Convulsion	Yes	No	Yes	No	No	No	No	Yes	Yes	No
Decreased LOC	Negative	Negative	Positive	Positive	Negative	Negative	Negative	Negative	Negative	Negative
Mechanical ventilation	Yes	Yes	Yes	Yes	No	Yes	Yes	Yes	Yes	Yes
ARDS classification	Severe	Moderate	Severe	Severe	Moderate	Severe	Severe	Moderate	Severe	Severe
History of contact	Negative	Positive	Positive	Negative	Positive	Negative	Negative	Positive	Positive	Negative
Died	Yes	No	Yes	No	No	Yes	Yes	No	No	Yes

*Immunodeficiency 10 is an autosomal recessive primary immunodeficiency characterised by the onset of recurrent infections in childhood due to defective T- and NK-cell function although the severity is variable.^22^

ARDS, acute respiratory distress syndrome; LOC, level of consciousness; UPJO, ureteropelvic junction obstruction.

**Table 3:** Laboratory results on day 1 of admission

COVID real-time PCR	Positive						Negative			
Patient	1	2	3	4	5	6	7	8	9	10
Ferritin (µg/L) M: 22.81–275 F: 4.63–204	580	1235	954	5701	750	1310	3489	1067	651	*
Typical chest CT finding	Positive	Positive	Positive	Positive	Positive	†	Positive	Positive	Positive	Positive
White cell count 109/L)	26 100	9800	7100	11 300	7500	22 000	14 000	12 000	3800	12 000
Lymphocyte	2088	1470	710	1017	1100	1100	6380	2400	760	480
Procalcitonin ≤0.3(µg/L)	0.24	30.5	10.1	1.36	0.6	*	1.8	73	0.2	*
C reactive protein <6 (mg/L)	32	90	50	150	37	5	3	85	3	3
Creatine phosphokinase (µkat/L) M:<2.86 F:<2.4	0.62	29.5	15.8	6.2	9.3	4.4	1.4	3.6	2.7	2.3
Lactate dehydrogenase (µkat/L)<8	10.6	10.6	94.5	30.3	22.5	22.5	19.2	13.3	27.8	15.6
Troponin (µg/L)<19	32	29.2	3343	2029	1.5	450	27	68	35	1.5
D-dimer (nmol/L) <2738	10 535	2957	54 760	50 554	7096	3993	28 119	52 219	7096	50 554
Total bilirubin (μmol/L) 5.1–17	11.9	5.1	598.6	10.2	6.8	3.4	*	5.1	10.2	*
Direct bilirubin (μmol/L) 3.4–12.0	5.1	1.7	359	1.7	3.4	1.7	*	1.7	1.7	*
Aspartate transaminase (µkat/L) M:<0.62 F:<0.52	17	0.6	33.9	0.85	0.67	1.6	*	1.2	1.1	*
Alanine aminotransferase (µkat/L) M:<0.68 F:<0.52	0.8	0.2	11.5	0.62	0.55	0.22	*	0.47	0.57	*
Creatinine (µmol/L) M: 53–106 F: 44–97	79.5	44.2	8.8	44.2	79.5	424.3	44.2	35.3	35.3	291.7
Blood urea nitrogen (mmol/L) 3.6–7.1	4.6	3.9	40.3	4.6	5	18.9	3.2	1	6.7	22.1

M: male/F: female.

*Did not check.

†Was not performed due to his unstable condition.

Five patients had a positive history of contact to confirmed or suspected cases (three patients had contact to positive RT-PCR cases) or possible cases with fever and cough. The main symptoms were fever, cough, abdominal pain, lethargy and encephalopathy. The median time from the presentation of symptoms to PICU admission was 4.7 days (range 3–8 days).

All the children had tachypnoea and tachycardia on admission to the PICU. All patients had Pa02/Fio2 (the ratio of arterial oxygen partial pressure to fractionalinspired oxygen) less than 300 and nine patients needed intubation and mechanical ventilation (all were intubated due to respiratory failure outside the PICU). One patient responded to oxygen supplementation via non-rebreathing mask despite decreased o2 saturation and PaO2 of 47 mm Hg.

In other intubated patients, the main problem was severe hypoxia. Although they needed a low peak inspiratory pressure for acceptable tidal volume, their oxygen saturation could not reach 85%, so peak expiratory pressure was increased even up to 18 and prone positioning was ordered to increase the oxygen saturation (cases number 4 and 6). Extracorporeal membrane oxygenation (ECMO) was not available in our centre.

Unfortunately, five patients died. Four of the five had chronic medical conditions. Cardiac arrhythmias occurred in three of the children who died.

Nine children had hypotension. Six children received high dose of norepinephrine (more than 0.3 μ/kg/min) in addition to other inotropes. Enoxaparin was started with the aim of antithrombotic prophylaxis in all the patients. In all patients broad spectrum antibiotics (meropenem and vancomycin) were started in addition to hydroxychloroquine (ECG was taken first and all had normal Corrected QT interval (QTc) in electrocardiogram; equal to or less than 0.40 s) and kaletra (lopinavir/ritonavir), and intravenous immune globulin was given to eight patients due to severe septic shock (except for cases number 5 and 10). In hypotensive patients, hydrocortisone, ascorbic acid and thiamine were started.

In all of the patients, blood culture, tracheal aspirate, urine culture and PCR for influenza A and B were negative (tables 2 and 3).

Patient number 3 presented with fever, abdominal pain and tachypnoea. He was transferred by his parents to our centre with the diagnosis of acute liver failure (Namazi Hospital is the referral centre for liver transplant in Iran). On arrival, he was intubated due to decreased o2 saturation and decreased level of consciousness. He had hypotension, fever and patchy ground glass infiltrations in the chest CT scan. Surprisingly, the serum bilirubin level was very high (total bilirubin was 35); the other lab data in addition to alkaline phosphatase: 387 and gamma-glutamyl transferase: 45 are shown in table 3. The patient tests for hepatitis A, B, C, Epstein-Barrvirus (EBV*),* herpessimplex virus(HSV), cytomegalovirus(CMV) were negative and serum ceruloplasmin level and anti-liver-kidney microsomal antibody were in normal range; he had a negative history of taking medications or substance abuse. His parents had fever and cough and their nasopharyngeal RT-PCR for COVID-19 turned positive in both.

## Discussion

There are few studies regarding paediatric patients admitted in PICU. Paediatric data from Madrid, Spain, reported no mortality, but described one child who needed mechanical ventilation and two who needed non-invasive ventilation.^11^ In a Chinese study, there was only one case who needed mechanical ventilation.^12^ In a cohort study in children hospital in Wuhan, three paediatric patients were admitted in the PICU, all with comorbidities and one case died.^13^ In another study from Wuhan, one patient without comorbidity was admitted in PICU and survived.^5^

In our study, all patients were febrile and 70% had cough, but in some studies, non-critical paediatric fever was present in less than 50%.^5 12 13^

In a study on 48 paediatric patients with COVID-19 admitted in 46 North American PICUs, 83% had significant pre-existing comorbidities, 73% presented with respiratory symptoms, 38% required invasive ventilation and 23% had failure of two or more organ systems. The mortality rate was 4% in their study (up to the time of the report). Three patients were intubated, one patient was taking ECMO and only 25% needed vasoactive support.^14^

In the beginning of the pandemic, it was assumed that the main organ involvement in COVID-19 was respiratory, but several studies have reported Kawasaki-like syndrome MIS-C later.^15^

DeBiasi *et al*^16^ described a 177 paediatric patients series in the Washington, DC metropolitan region; among them, 9 cases required critical care, 8 needed respiratory support and 1 had Kawasaki-like shock syndrome.

In our study, some of our severely infected patients had elevated troponin level and fulfilling the criteria for MIS-C on arrival to PICU. Also hypotension and organ hypoperfusion can explain the aetiology of high level of troponin.

It has been shown that coronavirus infection (SARS, Middle East Respiratory Syndrome and even; COVID-19) could damage the liver, and mild to moderate elevation of alanine aminotransferase (ALT); decreased albumin and increased serum bilirubin levels occur frequently,^17–20^ but extremely high ALT and bilirubin levels in patient number 3 was noteworthy with some probable explanation like acute liver failure caused by COVID-19 or a rare complication of the disease.^21^

Through reporting the characteristics of our patients, we aim to share our experience regarding COVID-19 patients.

There is a general conception that paediatric patients infected by COVID-19 have less severe symptoms and better outcomes, but severe and fatal cases occur as well.

## Supplementary Material

Author's manuscript
