# Supplementary material for: Clinical presentation of paediatric patients with COVID-19 admitted to a single paediatric intensive care unit (PICU) in Iran
Source: BMJ Paediatr Open. 2020 Sep 17;4(1):e000715. doi: 10.1136/bmjpo-2020-000715 (PMC7499680; doi:10.1136/bmjpo-2020-000715)
Supplement: Author's manuscript [file bmjpo-2020-000715.draft_revisions.pdf]

BMJ Paediatrics Open

BMJ Paediatrics Open is committed to open peer review. As part of this commitment we make the peer review history of every article we publish publicly available.

When an article is published we post the peer reviewers' comments and the authors' responses online. We also post the versions of the paper that were used during peer review. These are the versions that the peer review comments apply to.

The versions of the paper that follow are the versions that were submitted during the peer review process. They are not the versions of record or the final published versions. They should not be cited or distributed as the published version of this manuscript.

BMJ Paediatrics Open is an open access journal and the full, final, typeset and author-corrected version of record of the manuscript is available on our site with no access controls, subscription charges or pay-per-view fees (http://bmjpaedsopen.bmj.com).

If you have any questions on BMJ Paediatrics Open's open peer review process please email info.bmjpo@bmj.com

BMJ Paediatrics Open

Clinical presentation of pediatric patients with COVID 19 admitted in pediatric intensive care unit (PICU) in Iran

Journal:	*BMJ Paediatrics Open*
Manuscript ID	bmjpo-2020-000715
Article Type:	Original research
Date Submitted by the Author:	13-May-2020
Complete List of Authors:	Saeed, Amir<br>shorafa, eslam; Shiraz University of Medical Sciences,<br>sanaeidashti, anahita<br>kadivar, mohammad rahim
Keywords:	Health services research, Virology, Mortality

SCHOLARONE™
Manuscripts

I, the Submitting Author has the right to grant and does grant on behalf of all authors of the Work (as defined in the below author licence), an exclusive licence and/or a non-exclusive licence for contributions from authors who are: i) UK Crown employees; ii) where BMJ has agreed a CC-BY licence shall apply, and/or iii) in accordance with the terms applicable for US Federal Government officers or employees acting as part of their official duties; on a worldwide, perpetual, irrevocable, royalty-free basis to BMJ Publishing Group Ltd ("BMJ") its licensees and where the relevant Journal is co-owned by BMJ to the co-owners of the Journal, to publish the Work in this journal and any other BMJ products and to exploit all rights, as set out in our licence.

The Submitting Author accepts and understands that any supply made under these terms is made by BMJ to the Submitting Author unless you are acting as an employee on behalf of your employer or a postgraduate student of an affiliated institution which is paying any applicable article publishing charge ("APC") for Open Access articles. Where the Submitting Author wishes to make the Work available on an Open Access basis (and intends to pay the relevant APC), the terms of reuse of such Open Access shall be governed by a Creative Commons licence – details of these licences and which Creative Commons licence will apply to this Work are set out in our licence referred to above.

Other than as permitted in any relevant BMJ Author's Self Archiving Policies, I confirm this Work has not been accepted for publication elsewhere, is not being considered for publication elsewhere and does not duplicate material already published. I confirm all authors consent to publication of this Work and authorise the granting of this licence.

Confidential: For Review Only

Clinical presentation of pediatric patients with COVID 19 admitted in pediatric intensive care unit (PICU) in Iran

1.**Eslam Shorafa**

department of pediatrics, division of intensive care unit, Shiraz University of Medical Sciences, Shiraz, Iran.

E-mail; omidshorafa@gmail.com

Postal address: Khalili street, Namaze complex, apartment A, unit 3, Shiraz, iran

Phone number; +989363946906, fax number: +9836474298

2. **Amir Saeed**

department of pediatrics, division of intensive care unit, Shiraz University of Medical Sciences, Shiraz, Iran.

E-mail; dr.saeedamir@yahoo.com

3. **Anahita Sanaei Dashti**

professor of pediatric infectious diseases, professor Alborzi clinical microbiology research center, Shiraz University of Medical Sciences, Shiraz, Iran.

E-mail; anahitasanaei@gmail.com

4. **Mohammad Rahim Kadivar**

professor of pediatric infectious diseases, professor Alborzi clinical microbiology research center, Shiraz University of Medical Sciences, Shiraz, Iran.

E-mail; Kadmr1@gmail.com

Word count: 1522

Key word: COVID19; coronavirus; Wegener; PICU

Confidential: For Review Only

Abstract

Objectives: To describe clinical characteristics and laboratory data of pediatric patients who were admitted in pediatric intensive care unit (PICU) with the impression of COVID-19.

Design: A cross sectional study

Setting and participants: Ten pediatric patients admitted in PICU from 16th of March, 2020 to 21th of April, 2020, were enrolled in this study

Main outcome measures: Clinical and para clinical characteristics and outcome of the patients

Result: The median age was 7.9 year, the youngest was 4 months old and the oldest was 16 years old and two of them were female .The most common symptoms were; fever and cough. The most common reason for PICU admission was respiratory failure (in 9 patients). In half of patients, there was a history of contact to confirmed or suspicious people, six patients had pre-existing medical condition. Most of the patients had hypotension and received inotrope and six patients needed norepinephrine infusion added to dopamine and epinephrine.

Of all, 5 patients died and the most common cause of it, was refractory cardiac arrhythmia

Conclusion: COVID-19 can be fatal in pediatric patients especially in those who have a comorbidity or underlying disease and those who have delayed medical referral and or management.

It seems prudent to choose a more valid stricter triage criteria for pediatric emergency rooms and educate the physicians and nurses involving in pediatric care to better and early diagnose COVID 19 cases in this age group.

What is known about the subject?

► COVID -19 infection in is less frequent and severe in pediatric patients than adult patients.

► The main symptom is fever and few pediatric patients need mechanical ventilation or inotrope support.

Confidential: For Review Only

What this study adds?

► COVID -19 infection could be fatal pediatric patients especially in those who have comorbidities.

►The main respiratory problems in severe cases are oxygenation failure and in cardiovascular; hypotensive state necessitating inotropes.

► The main causes of mortality in our study were cardiac arrhythmia and refractory hypoxemia.

Confidential: For Review Only

Introduction

In December 2019, a cluster of acute respiratory illness occurred in Wuhan, Hubei Province, China by novel coronavirus named severe acute respiratory syndrome coronavirus-2 (SARS-CoV-2) (1). On 3rd of January 2020, the2019novel coronavirus (2019-nCoV) was identified in samples of bronchoalveolar lavage fluid from a patient in Wuhan and was confirmed as the cause of that (2).

The current outbreak of infections with SARS-CoV-2 is termed Coronavirus

Disease 2019 (COVID-19) by the World Health Organization (WHO) (3). The disease has rapidly spreads from Wuhan to other areas and worldwide so that world health organization (WHO) in 11th march 2020 announces COVID-19 outbreak a pandemic (4).

The 2019-nCoV has close similarity to bat coronaviruses, and it has been assumed that bats are the primary source. While the origin of the 2019-nCoV is still being investigated, current evidence suggests spread to humans occurred via transmission from wild animals illegally sold in the Huanan Seafood Wholesale Market.

The rate of infection with covid-19 in children is low and the first pediatric case was reported on January 20, 2020; was a 10-year-old Chinese boy, whose family had visited Wuhan City (4). A retrospective study was done in 366 children whose ages were ≤16 years old and were hospitalized for respiratory infections between January 7 and 15, 2020, COVID-19 was confirmed in 6 (1.6%) children whose onset of illness occurred between January 2 and 8, 2020. This study results suggests that COVID-19 infections in children were occurring early in the epidemic (5) One reason of low incidence rate in children in China is that; children were rarely tested for the virus in earlier phase of the outbreak, especially in Hubei province, where the most patients were confirmed so we have a limited data on the prevalence of COVID-19 in children (6).

In Iran, the first cases detected in February 2020 in Qom city and from there, the disease spread to whole country. In critically ill pediatric patients, also there are a few reports.

In this article we want to describe the characteristics of pediatric patients with COVID-19 admitted in pediatric intensive care unit (PICU) of Namazi hospital in Shiraz, Iran.

Patients and Method

From March 16th to April 21th, the patients admitted in PICU of Namazi hospital in Shiraz, Iran (Namazi Hospital ;the largest hospital in south of Iran with 750 beds is located in Shiraz city that is the main tertiary referral center in south of Iran which has 18 beds general PICU and 9 beds surgery PICU);and those whom with laboratory data and Chest CT scan COVID-19 confirmed for them or according to clinical presentation and chest CT scan were highly suspicious to COVID-19, included in this study. A laboratory confirmed case of Covid-19 was defined by a positive result on a reverse transcriptase–polymerase chain-reaction (RT-PCR) assay of a specimen collected on an oropharyngeal swab, nasopharyngeal swab or bronchoalveolar lavage. The patients whose ages were between one month to 18 year old included in this study. The patients younger than one month and older than 18 year old excluded from this study.

Patient involvement

Patients were not directly involved in the design of this study.

Confidential: For Review Only

Ethics statement

This study was approved by the ethics committee of Shiraz University of Medical science (IR.sums.med.rec.1399.40). Written informed consent was obtained the parents guardians and sent to the ethics committee.

Results

From 16th of march to 21th of April, 2020, ten patients admitted in PICU and COVID-19 diagnosed for them by RT-PCR or laboratory data and chest CT scan findings (Figure 1,2). In those, six patients had positive RT-PCR for COVID-19 and others had positive chest CT Scan findings and laboratory data in favor of COVID-19. The median age was 7.9 years and the youngest was four months old and the oldest was 16 year old. Eight patients were male. Six patients had underlying disease and four were healthy children before the PICU admission. Five patients had positive history of contact to confirmed or suspicious persons. The main symptoms were: fever, cough, abdominal pain, lethargy and encephalopathy. Four patients developed with convulsion during PICU stay (patients number: 2, 3,4 and 6) and in two patients, brain CT scan revealed severe brain edema (patents number: 2 and 3).

Unfortunately five of 10 patients expired (patients number:1, 2, 6, 9 and 10) ; of those, four patients had comorbidity before admission and in three patients cardiac arrhythmia was the cause of death (patients number 2, 6 and 9), and the last two was due to refractory hypoxia(case number 1,10).

The median time (day) from the presentation of symptoms to PICU admission was 4.7days, the minimum was 3 days and maximum was 8 days and that was in a patient who due to fear of COVID-19 infection, refused to go to hospital.

Four patients (cases number:2, 4, 6 and 9) developed with cardiac arrhythmia so that in three patients(patients number:2, 6, and 9) were refractory to medical therapy and resulted in cardiac arrest .

All patients had Pa02/Fio2 less than 300 and of all ,nine patients needed intubation and mechanical ventilation, and in one patient(patient number:8) despite of decreased o2 saturation and Pa02;47 mmhg ,he responded to o2 via non rebreathing mask, in the others whom were intubated , their main problem was severe hypoxia but although they needed low peak pressure(by decreasing peak inspiratory pressure(PIP) for enough tidal volume, and respiratory rate(unlike the usual cases we have seen in ARDS), o2 saturation could not reach

Confidential: For Review Only

85 so that needed increasing peak expiratory pressure(PEEP) up to 18 and prone positioning.

Of nine patients admitted only two patients didn't have hypotension and of eight patients developed with hypotension six patients had vasoplegic shock, so that needed infusion of high dose norepinephrine.

In one patient (patient number 4) in order to close and continuous monitoring of hemodynamic and volume status of patient, in addition to bedside echocardiography that was done for all patients, arterial line inserted and monitored by proAQT device.

In all patients broad spectrum antibiotic started in addition to hydroxychloroquine and kaletra (lopinavir/ritonavir), but in 7 patients intravenous globulin (IVIG) (except cases no: 8 and 10) was given. In hypotensive patients, hydrocortisone, ascorbic acid and thiamine started.

In all of the patients, blood culture, tracheal aspirate culture and urine culture (especially for patient number 2 that was done two times) were negative, and also PCR for influenza A and B, in all patients were negative (Table 1, 2).

In patient number 6; the patient presented with fever, abdominal pain and tachypnea (both of his parents had fever and cough and in our center RT-PCR was done for them and both were positive). His symptoms progressed to lethargy so admitted in a hospital in Babol(a city in north of Iran which in that time had a surge in COVID-19 infection) seven days prior to our center's PICU admission and in that hospital, his condition deteriorated and developed icter so with the diagnosis of acute liver failure transferred to our center. On arrival, due to decreased o2 saturation and decreased level of consciousness, he was intubated and transferred to PICU. On PICU arrival, the patient had hypotension, fever and in chest CT scan had patchy infiltration. All lab data could be related to COVID-19 except high bilirubin that has not seen as high as our patient was (total bilirubin was 35) in COVID-19 (The patient tested negative for hepatitis A, B, C, EBV, CMV and serum ceruloplasmin was in normal range).

In patient number 2, abdominal sonography revealed no abscess formation or collection.

Confidential: For Review Only

COVID Real-time	positive	negative

Table 1: Epidemiologic & Clinical findings of the patients

Patient number	1	2	3	4	5	6	7	8	9	10
Age(years)	4 months	1	1.5	3	3	11	13	15	16	16
Sex	male	male	female	female	male	male	male	male	male	male
Hypotension	Yes	Yes	Yes	Yes	Yes	Yes	Yes	No	Yes	Yes
Tachycardia on admission	Yes	Yes	Yes	Yes	Yes	Yes	Yes	Yes	Yes	Yes
Tachypnea on admission	Yes	Yes	Yes	Yes	Yes	Yes	Yes	Yes	Yes	Yes
Chief complaint(s)	Fever & Letharginess	Fever & Letharginess	Fever & cough	Fever & cough	Fever & cough	Fever & abdominal pain & decreased LOC	Fever & Letharginess	Fever & cough	Fever & cough	Fever & cough
Previous illness	Cerebral Shunt	UPJO• with nephrostomy	negative	Immunodeficiency	negative	negative	Cerebral palsy	negative	wegener granolomatosis	Cerebral palsy
Convulsion	Yes	No	Yes	Yes	No	Yes	No	No	No	No
Decreased LOC*	negative	negative	negative	negative	negative	positive	positive	negative	negative	negative
Mechanical ventilation	Yes	Yes	Yes	Yes	Yes	Yes	Yes	No	Yes	Yes
ARDS# classification	Severe	Severe	moderate	Severe	moderate	Severe	Severe	Moderate	Severe	Severe
History of contact	negative	negative	positive	positive	positive	positive	negative	positive	negative	negative
Died	Yes	Yes	No	No	No	Yes	No	No	Yes	Yes

*LOC: level of consciousness

#ARDS: Acute respiratory distress syndrome

• UPJO: urethropelvic junction obstruction

Confidential: For Review Only

PCR										
Patient	1	5	6	7	8	9	2	3	4	10
Ferritin (ng/ml) M: 22.81-275 F: 4.63-204	580	1235	954	5701	750	1310	3489	1067	651	*
Typical Chest CT finding	positive	positive	positive	positive	positive	#	positive	positive	positive	positive
White blood cells(count/ml)	26100	9800	7100	11300	7500	22000	14000	12000	3800	12000
Lymphocyte	2088	1470	710	1017	1100	1100	6380	2400	760	480
Procalcitonin ≤ 0.3	0.24	30.5	10.1	1.36	0.6	*	1.8	73	0.2	*
C- reactive protein <6 (mg/L)	32	90	50	150	37	5	3	85	3	3
Creatine phosphokinase (U/L) M: < 171 F:< 145	37	1769	950	374	557	264	88	220	163	140
Lactate dehydrogenase (U/L) < 480	640	635	5660	1820	1350	1350	1150	786	1670	938
Troponin (ng/ml) < 19	32	29.2	3343	2029	1.5	450	27	68	35	1.5
D-Dimer (ng/ml) < 500	1924	540	10000	9232	1296	638	5135	9536	1296	10000
Total bilirubin 0.1-1.2	0.7	0.3	35	0.6	0.4	0.2	*	0.3	0.6	*
Direct bilirubin < 0.3	0.3	0.1	21	0.1	0.2	0.1	*	0.1	0.1	*
Aspartate transaminase (U/L) M: < 37 F: < 31	1020	36	2030	51	40	97	*	77	68	*
Alanine aminotransferase (U/L) M: < 41 F: < 31	52	14	690	37	33	13	*	28	34	*
Creatinine M: 0.8 - 1.3 F: 0.6 - 1.2	13	0.5	0.1	0.5	0.9	4.8	0.5	0.4	0.4	3.3
Blood urea nitrogen (mg/dl) 8 - 20	0.9	11	113	13	14	53	9	3	19	62

Table 2: Para clinical findings on day one of admission

M: male/F: female

did not performed due to his unstable condition

*did not checked

Confidential: For Review Only

Discussion

The overall case fatality rate as per China Centre for Disease Control and Prevention (CDC) is 2.3%, which is much lower compared to SARS (9.6%) and MERS (34%) but significantly higher compared to the latest H1N1 influenza pandemic (0.001 – 0.007%) (7). However, as per WHO, the global case.

Fatality rate is as high as 4.4% with absolute number of deaths already higher than the total fatality of SARS and MERS combined (8). The case fatality reported from Italy is 7.2% which has gone up to 9.8% as per WHO (as on March 26, 2020) (9).

In pediatric patients admitted in PICU, we have limited data, The case fatality rate in our PICU was 50% and in adult patients admitted in ICU is about 50% (10,11,12) but in limited data we have; in Madrid, Spain, in a study there was no mortality(13) and in a Chinese study one patient(14). In a cohort study in Wuhan children hospital; three patients admitted in PICU and one of three (33%) died and all the patients had comorbidities (15). In another study in Wuhan one patient without comorbidity admitted in PICU and survived (5).

In our study, all patients were febrile and 70% had cough but in some studies done in non-critical pediatric patients were less than 50 %(5, 14, 15).

In our study, 60% patients had positive RT-PCR FOR COVID-19, that there are some reasons for that; first is in the first day we tested the patients via oropharyngeal swab and the other possibility is that the patients arrived PICU had severe disease and were in terminal stage of disease.

It has been shown that in coronavirus infection (SARS, MERS and even;COVID-19)liver can be damaged and we see increased Alanine aminotransferase (ALT) , decreased albumin and increased serum bilirubin levels but have seen mild to moderate elevation(16,17,18,19) in one of our patient(number:6) , we saw increased ALT and bilirubin, but they were much more than the other patients . So there are some possibilities for that: acute liver failure with COVID-19 infection, rare complications of COVID-19…

Confidential: For Review Only

Conclusion

There is a general conception that pediatric patients infected by COVID-19,it has less severe symptoms and better outcome but we should consider the group that has comorbidity has a higher risk of infection and worse outcome and in this people ,we should consider routine screening test and education of warning signs and symptoms and using personal protective equipment . Even in general pediatric population whom taken to emergency rooms and physician, we should consider a stricter triage for screening the ill pediatric patients.

Conflict of interest: none

Funding: The authors have not declared a specific grant for this research from any funding agency in the public, commercial or not-for-profit sectors.

Authors' Contributions: A.S. planned the study and wrote the manuscript. E.S. gathered patients' data and submitted the manuscript. A.S.D. data interpretation .A.S.D. and M.R.K. edited the manuscript and were scientific consultant .All authors discussed the results and contributed to the final manuscript.

Confidential: For Review Only

References

1. Lu H, Stratton CW, Tang YW. Outbreak of pneumonia of unknown etiology in Wuhan China: the mystery and the miracle. J Med Virol 2020; 92:401-2.

2. Zhu N, Zhang D, WangW, et al; China Novel Coronavirus Investigating and Research Team. A novel coronavirus from patients with pneumonia in China, 2019 [published January 24, 2020]. N Engl JMed.doi:10.1056/NEJMoa2001017

3. World Health Organization. Situation Report 1 2020 (World Health Organization. Novel coronavirus (2019-nCoV), situation report-1. 21 January 2020 [Internet]. Geneva

4. Chan JF, Yuan S, Kok KH, To KK, Chu H, Yang J, et al. A familial cluster of pneumonia associated with the 2019 novel coronavirus indicating person-to-person transmission: a study of a family cluster. Lancet. 2020;395(10223):514-23.

5. Liu W, Zhang Q, Chen J, Xiang R, Song H, Shu S, et al. Detection of Covid-19 in Children in Early January 2020 in Wuhan, China. N Engl J Med. 2020.

6. Cao Q, Chen YC, Chen CL, Chiu CH. SARS-CoV-2 infection in children: Transmission dynamics and clinical characteristics. J Formos Med Assoc. 2020.

7. Wu Z, McGoogan JM. Characteristics of and important lessons from the coronavirus disease 2019 (COVID-19) outbreak in China. JAMA. Published online February 24, 2020. doi:10.1001/jama.2020.2648.

8. Mahase E. Coronavirus: covid-19 has killed more people than SARS and MERS combined, despite lower case fatality rate. BMJ. 2020; 368:m641.

9. Onder G, Rezza G, Brusaferro S. Case-fatality rate and characteristics of patients dying in relation to COVID-19 in Italy. JAMA. Published online March 23, 2020. doi:10.1001/jama.2020.4683.

10. Arentz M, Yim E, Klaff L, et al. Characteristics and outcomes of 21 critically ill patients with COVID-19 in Washington State. JAMA 2020 March 19 (Epub ahead of print).

11. Guan W, Ni Z, Hu Y, et al. Clinical characteristics of coronavirus disease 2019 in China. N Engl J Med. DOI: 10.1056/ NEJMoa2002032.

Confidential: For Review Only

12. Bhatraju, P. K., et al. (2020). "Covid-19 in Critically Ill Patients in the Seattle Region — Case Series." New England Journal of Medicine.

13. Tagarro, A., et al. (2020). "Screening and Severity of Coronavirus Disease 2019 (COVID-19) in Children in Madrid, Spain." JAMA Pediatr.

14. Lu, X., et al. (2020). "SARS-CoV-2 Infection in Children." New England Journal of Medicine.

15. Dong Y, Mo X, Hu Y, et al: Epidemiological characteristics of 2143 pediatric patients with 2019 coronavirus disease in China. Pediatrics 2020 Mar 16. [Online ahead of print]

16. Lu S-N, Lee C-M, Lin M-C, et al. Sequential changes of serum aminotransferase levels in patients with severe acute respiratory syndrome.

Am J Trop Med Hyg. 2004; 71(2):125-128.

17. Arabi YM, Al-Omari A, Mandourah Y, et al. Critically Ill patients with the Middle East respiratory syndrome. Crit Care Med.

2017; 45(10):1683-1695.

18. Xu, L, Liu, J, Lu, M, Yang, D, Zheng, X. Liver injury during highly pathogenic human coronavirus infections. LiverInt. 2020; 00: 1 7. https://doi.org/10.1111/liv.14435

19. Huang C, Wang Y, Li X, et al. Clinical features of patients infected with 2019 novel coronavirus in Wuhan, China. Lancet 2020; 395: 497–506.

Confidential: For Review Only

Confidential: For Review Only

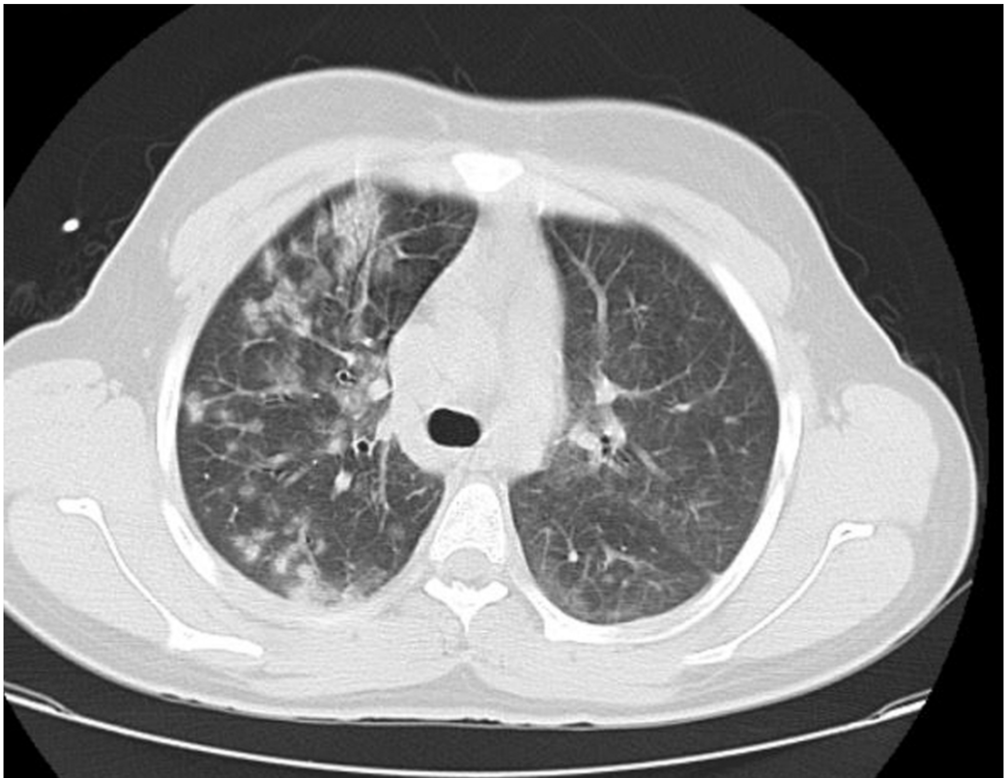

Figure 1: Chest ct of patient number 1

99x79mm (300 x 300 DPI)

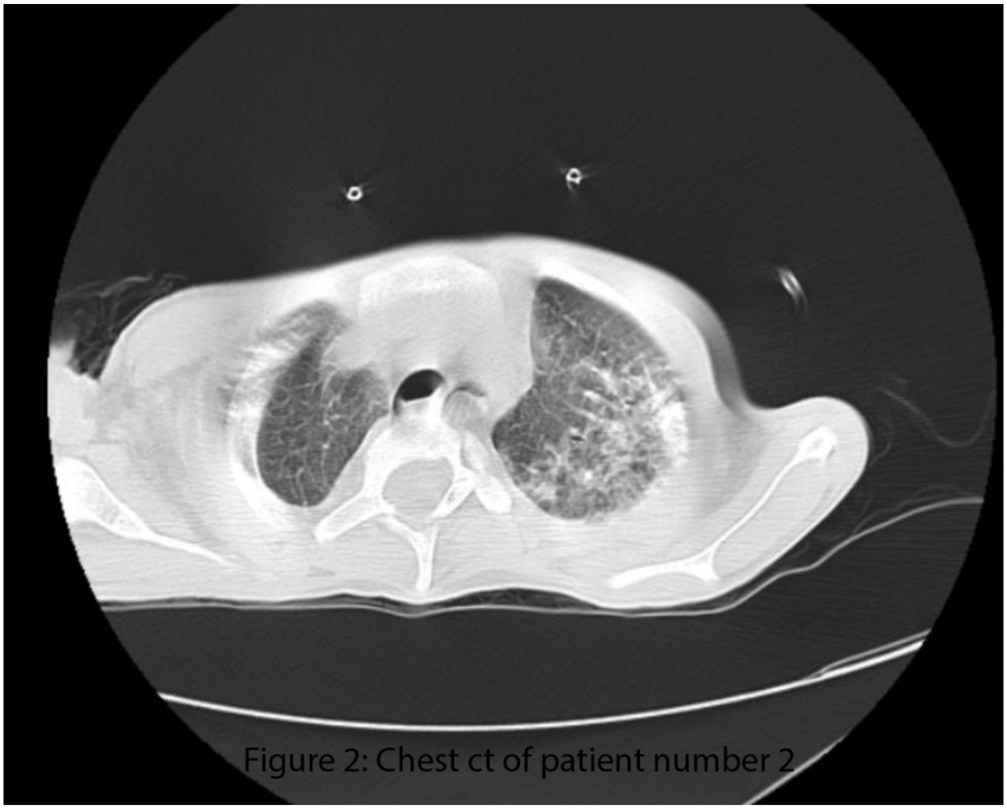

Figure 2: Chest ct of patient number 2

99x79mm (300 x 300 DPI)

BMJ Paediatrics Open

Clinical presentation of pediatric patients with COVID 19 admitted in a single pediatric intensive- care unit (PICU) in Iran

Journal:	*BMJ Paediatrics Open*
Manuscript ID	bmjpo-2020-000715.R1
Article Type:	Original research
Date Submitted by the Author:	08-Jul-2020
Complete List of Authors:	Saeed, Amir; Shiraz University of Medical Sciences Medical School, Pediatrics<br>shorafa, eslam; Shiraz University of Medical Sciences,<br>sanaeidashti, anahita; Shiraz University of Medical Sciences, department of pediatrics<br>kadivar, mohammad rahim; Shiraz University of Medical Sciences, department of pediatrics
Keywords:	Health services research, Virology, Mortality

SCHOLARONE™
Manuscripts

BMJ

I, the Submitting Author has the right to grant and does grant on behalf of all authors of the Work (as defined in the below author licence), an exclusive licence and/or a non-exclusive licence for contributions from authors who are: i) UK Crown employees; ii) where BMJ has agreed a CC-BY licence shall apply, and/or iii) in accordance with the terms applicable for US Federal Government officers or employees acting as part of their official duties; on a worldwide, perpetual, irrevocable, royalty-free basis to BMJ Publishing Group Ltd ("BMJ") its licensees and where the relevant Journal is co-owned by BMJ to the co-owners of the Journal, to publish the Work in this journal and any other BMJ products and to exploit all rights, as set out in our licence.

The Submitting Author accepts and understands that any supply made under these terms is made by BMJ to the Submitting Author unless you are acting as an employee on behalf of your employer or a postgraduate student of an affiliated institution which is paying any applicable article publishing charge ("APC") for Open Access articles. Where the Submitting Author wishes to make the Work available on an Open Access basis (and intends to pay the relevant APC), the terms of reuse of such Open Access shall be governed by a Creative Commons licence – details of these licences and which Creative Commons licence will apply to this Work are set out in our licence referred to above.

Other than as permitted in any relevant BMJ Author's Self Archiving Policies, I confirm this Work has not been accepted for publication elsewhere, is not being considered for publication elsewhere and does not duplicate material already published. I confirm all authors consent to publication of this Work and authorise the granting of this licence.

Confidential: For Review Only

Clinical presentation of pediatric patients with COVID 19 admitted in a single pediatric intensive care unit (PICU) in Iran

1. **Eslam Shorafa**

Assistant Professor of Pediatric Intensive Care, Department of Pediatrics, Division of Intensive Care Unit, Shiraz University of Medical Sciences, Shiraz, Iran.

E-mail; omidshorafa@gmail.com

Postal address: Khalili street, Namazi complex, apartment A, unit 3, Shiraz, Iran

Phone number; +989363946906, fax number: +9836474298

2. **Amir Saeed**

Assistant Professor of Pediatric Intensive Care, Department of Pediatrics, Division of Intensive Care Unit, Shiraz University of Medical Sciences, Shiraz, Iran.

E-mail; dr.saeedamir@yahoo.com

3. **Anahita Sanaei Dashti**

Professor of Pediatric Infectious Diseases, Professor Alborzi Clinical Microbiology Research Center, Shiraz University of Medical Sciences, Shiraz, Iran.

E-mail; anahitasanaei@gmail.com

4. **Mohammad Rahim Kadivar**

Professor of Pediatric Infectious Diseases, Professor Alborzi Clinical Microbiology Research Center, Shiraz University of Medical Sciences, Shiraz, Iran.

E-mail; Kadmr1@gmail.com

Word count: 1522

Keywords: COVID 19; SARS coronavirus 2 , Wegener , child, PICU

Confidential: For Review Only

Abstract

Objectives: To describe the clinical manifestation, disease course, complications, co-morbidities, organ involvements apart from the lungs, outcome, and paraclinical characteristics of pediatric patients admitted in a single pediatric intensive care unit (PICU) in Iran with the impression of COVID-19

Design: A cross- sectional study

Setting and participants: Ten pediatric patients who were admitted in PICU from March 16 to April 21, 2020 . were enrolled in this study

Main outcome measures: Clinical and para-clinical characteristics, complications, and outcome of the patients

Result: The participants' median age was 7.9 years. The youngest one was 4 months old and the oldest 16 years old; two of them were female. The most common symptoms were fever and cough. Also, the most common reason for PICU admission was respiratory failure (in nine patients). In half of the patients, there was a history of contact to confirmed or suspicious COVID 19 cases. Six patients had a pre-existing medical condition. Most of the patients had hypotension on admission and received inotrope; six of them received norepinephrine infusion added to dopamine and epinephrine. Of them, 5 patients died and its most common cause was refractory cardiac arrhythmia.

Conclusion: COVID-19 can be fatal in pediatric patients, especially in those with a comorbidity or underlying disease and those who have delayed medical referral and or management.

What is known about the subject?

► COVID -19 infection is less frequent and severe in pediatric patients than adult patients.

► The main symptom is fever and few pediatric patients need mechanical ventilation or inotrope support.

Confidential: For Review Only

What this study adds?

► COVID-19 infection could be fatal in pediatric patients, especially in those with comorbidities.

►The main respiratory problems in severe cases are oxygenation failure and a hypotensive state necessitating inotropes.

► The main causes of mortality in our study were cardiac arrhythmia and refractory hypoxemia.

Introduction

In December 2019, a cluster of acute respiratory illness occurred in Wuhan, Hubei Province, China by a novel coronavirus named severe acute respiratory syndrome coronavirus-2 (SARS-CoV-2) (1). On the 3rd of January 2020, the virus was identified in the samples of bronchoalveolar lavage fluid from a patient in Wuhan and was confirmed as the cause of the disease (2).

The current outbreak of infections with SARS-CoV-2 was termed Coronavirus Disease 2019 (COVID-19) by the World Health Organization (WHO) (3). The disease rapidly spread from Wuhan to other areas of the world, so that the world health organization (WHO) announced Covid-19 outbreak as a pandemic on March 11, 2020 (4).

The 2019-nCoV is closely similar to bat coronaviruses, and it has been assumed that bats are the primary source. While the origin of the 2019-nCoV is still being investigated, the current evidence suggests that spread to humans occurred from wild animals illegally sold in the Huanan Seafood Wholesale Market.

The rate of infection with covid-19 in children is low and the first pediatric case was reported on January 20, 2020, who was a 10-year-old Chinese boy, whose family had visited Wuhan City (4). A retrospective study on 366 children aged

Confidential: For Review Only

under 16 years old and hospitalized for respiratory infections between January 7 and 15, 2020, confirmed COVID-19 infection in six (1.6%) of them. This study suggests that COVID-19 infections in children occurred early in the epidemic (5). One reason for the low frequency of the disease in children in China could be the fact that children were rarely tested for the virus in the earlier phase of the outbreak, especially in Hubei province, where most of the patients were confirmed, so there are a limited data on the prevalence of COVID-19 in children (6). The virus can produce a Kawasaki-like illness in children. World health organization (WHO) developed a preliminary case definition for this condition which was later named 'multisystem inflammatory disorder' in COVID-19 (MIS-C). The syndrome should be considered in children with features of typical or atypical Kawasaki disease or toxic shock syndrome.

Preliminary case definition:

Children and adolescents 0-19 years of age with fever > 3 days

AND two of the following:

a) Rash or bilateral non-purulent conjunctivitis or muco-cutaneous inflammation signs (oral, hands, or feet)

b) Hypotension or shock

c) Features of myocardial dysfunction, pericarditis, valvulitis, or coronary abnormalities (including ECHO findings or elevated Troponin/NT-proBNP)

d) Evidence of coagulopathy (by PT, PTT, and elevated d-Dimers)

e) Acute gastrointestinal problems (diarrhea, vomiting, or abdominal pain)

AND

Elevated markers of inflammation such as ESR, C-reactive protein, or procalcitonin

AND

No other obvious microbial cause of inflammation, including bacterial sepsis, staphylococcal or streptococcal shock syndromes

AND

Confidential: For Review Only

Evidence of COVID-19 (RT-PCR, antigen test or serology positive), or likely contact with patients with COVID-19(7).

Herein, we describe the patients with COVID-19 presenting with multi-system inflammatory syndrome. In Iran, the first COVID-19 cases were detected in February 2020 in Qom city. There are a few reports from critically ill pediatric patients in the country. In this article, we describe the characteristics of pediatric patients with COVID-19 admitted in pediatric intensive care unit (PICU) of Namazi hospital in Shiraz, Iran.

Patients and Method

From March 16 to April 21, the patients who were admitted in PICU of Nemazi hospital in Shiraz, Iran, were enrolled in the study. Nemazi educational hospital is the largest and the main tertiary referral center in the south of Iran with more than one thousand beds. The medical PICU of this hospital has 18 beds with two separate sections, one of which was devoted to COVID 19 cases from the beginning of the outbreak and those with suspicious or confirmed COVID-19 were admitted in this ward. A confirmed case of Covid-19 was defined by a positive result on a reverse transcriptase–polymerase chain-reaction (RT-PCR) assay of a specimen collected on an oropharyngeal swab, nasopharyngeal swab or bronchoalveolar lavage. The tests were performed using an Applied Biosystem Step One plus real-time PCR machine (Applied Biosystem, CA, USA). Amplification of N and ORF1b took place in a 20μL single-tube, and Superscript III Platinum one-step quantitative RT-PCR system (Invitrogen, Carlsbad, CA). Reactions contained 10.0 μL of 2X RT/PCR reaction mix, 1 μL primers/prob mix, 0.4 μL Superscript III RT/Platinum Taq mix, 0.4 μL ROX reference dye, and 5 μL of extracted sample RNA or serially diluted previously confirmed patients' positive control. The cycling conditions consisted of one cycle at 50°C for 10 min, one cycle at 95°C for 2 min, 45 cycles at 95°C for 5 s, and 60°C for 30 min)(8).

A suspicious case of COVID 19 was defined as a patient with clinical, laboratory findings (9) and chest CT findings consistent with COVID 19 infection (10), but negative PCR result. The patients whose ages were between one month to 18-years old who were admitted in PICU were enrolled in this study. The patients younger than one month and older than 18 years old were excluded from the study.

Patient involvement

Confidential: For Review Only

The patients were not directly involved in the design of this study.

Ethics statement

This study was approved by the ethics committee of Shiraz University of Medical Sciences (IR.sums.med.rec.1399.40). Written informed consents were obtained from the parents and sent to the ethics committee.

Results

From March 16 to April 21, 2020, ten patients were admitted in PICU with the impression of COVID-19 (Tables 1,2). Nine of them had been admitted in pediatric wards with impressions other than COVID-19 before transfer to PICU and only one of them was transferred from emergency room (ER) with the impression of COVID-19. From them, six patients had positive RT-PCR for COVID-19 and the others were suspicious with COVID-19. The median age was 7.9 years; the youngest one was four and the oldest was 16 years old. Eight patients were male. Also, six patients had underlying disease (Table 1) and four were already healthy children. Five patients had a positive history of contact to confirmed or suspicious persons (3 patients had contact to positive RT-PCR cases) or suspicious persons with fever and cough. The main symptoms were fever, cough, abdominal pain, lethargy and encephalopathy.

Four patients developed convulsion during PICU stay (patients number: 3, 7, 8 and 9) and brain CT of two of them revealed severe brain edema (patients number: 7 and 8); (other possible causes of decreased level of consciousness and convulsion, such as trauma, poisoning, electrolyte imbalance, etc. were evaluated and ruled out).

Unfortunately, five out of 10 patients passed away (patients number: 1,3, 6,7, and 10); of them, four patients had comorbidity before admission and in three patients cardiac arrhythmia was the cause of death (patients number 3,6 and 7). The median time from the presentation of symptoms to PICU admission was 4.7 days; the minimum was 3 days and maximum was 8 days (occurring in a patient who refused to come to hospital due to fear of COVID-19 infection (case 6)).

Four patients (cases: 3,6,7 and 9) developed cardiac arrhythmia that was refractory to medical therapy and resulted in ventricular fibrillation and cardiac arrest; patients number 3 and 6 had severe hypoxia (po2<50) at the presenting time of cardiac arrhythmia.

Confidential: For Review Only

All patients had Pa02/Fio2 less than 300 and nine patients needed intubation and mechanical ventilation (all were intubated due to respiratory failure outside the PICU). One patient (number 5) responded to oxygen supplementation via non-rebreathing mask despite decreased o2 saturation and PaO2 of 47 mmHg. In other intubated patients, the main problem was severe hypoxia. Although they needed a low peak inspiratory pressure (PIP) for acceptable tidal volume, their oxygen saturation could not reach 85% , so peak expiratory pressure (PEEP) was increased even up to 18 and prone positioning was ordered to increase the oxygen saturation. (cases number 4 and 6). It should be mentioned that we do not have extracorporeal membrane oxygenation (ECMO) in our center.

For close and continuous monitoring of hemodynamic and volume status, the arterial line was inserted in one patient (patient number 9) and monitored by proAQT device. Bedsides, echocardiography was done for all patients. Of ten patients admitted, only two patients did not have hypotension and of eight patients with hypotension, six had vasoplegic shock, so infusion of high dose norepinephrine was added duo to hypotension that was refractory to epinephrine/dopamine infusion; also, in patient number 9, Systemic Vascular Resistance Index (SVRI) was low (673)(dyn*s/cm5*m2) (reference range 1700-2400 according to proAQT device normal range). Enoxaparin was started with the aim of antithrombotic prophylaxis in all the patients.

In all patients broad spectrum antibiotics (meropenem and vancomycin) were started in addition to hydroxychloroquine (EKG was taken first and all had normal QTC; equal to or less than 0.40s) and kaletra (lopinavir/ritonavir), and intravenous immune globulin was given (IVIG) to 8 patients due to severe septic shock (except for cases number 5 and 10). In hypotensive patients, hydrocortisone, ascorbic acid, and thiamine were started.

In all of the patients, blood culture, tracheal aspirate, urine culture, and PCR for influenza A and B were negative (Table 1, 2).

Patient number 3 presented with fever, abdominal pain and tachypnea in Babol (a city in the north of Iran which had a surge in COVID-19 cases at that time). He had been admitted in Babol city hospital seven days prior to admission in our center. During the hospital stay, his condition deteriorated and he developed icterus, so he was taken by his parents' vehicle to our center (it's about 14 hours from our center) with the diagnosis of acute liver failure (Nemazi hospital is the referral center for liver transplant in Iran). On arrival, he was intubated due

Confidential: For Review Only

to decreased o2 saturation and decreased level of consciousness. He had hypotension, fever and patchy ground glass infiltrations in the chest CT scan. Surprisingly, the serum bilirubin level was very high (total bilirubin was 35); the other lab data in addition to alkaline phosphatase:387 and Gamma-glutamyl transferase (GGT) was:45 are shown in Table 1.The patient tests for hepatitis A, B, C, EBV, HSV,the and CMV were negative and serum ceruloplasmin level and LKM Ab were in normal range; he had a negative history of taking medications or substance abuse. His parents had fever and cough and their nasopharyngeal RT-PCR for COVID 19 turned positive in both.

In patient number 7, abdominal sonography revealed no abscess formation or collection.

Confidential: For Review Only

Table 1: Epidemiologic and clinical findings of the patients

Patient number	1	2	3	4	5	6	7	8	9	10
Age(years)	4 months	3	11	13	15	16	1	1.5	3	16
Sex	male	male	male	male	male	male	male	female	female	male
Hypotension	Yes	Yes	Yes	Yes	No	Yes	Yes	Yes	Yes	Yes
Tachycardia on admission	Yes	Yes	Yes	Yes	Yes	Yes	Yes	Yes	Yes	Yes
Tachypnea on admission	Yes	Yes	Yes	Yes	Yes	Yes	Yes	Yes	Yes	Yes
Chief complaint(s)	Fever & Letharginess	Fever & cough	Fever & abdominal pain & decreased LOC	Fever & Lethar giness	Fever & cough	Fever & cough	Fever & Lethargi ness	Fever & cough	Fever & cough	Fever & cough
Previous illness	Cerebral Shunt	negati ve	negative	Cerebr al palsy	negative	wegener granolom atosis	UPJO• with nephrost omy	negative	Immuno deficien cy∞	Cerebral palsy
Convulsion	Yes	No	Yes	No	No	No	No	Yes	Yes	No
Decreased LOC*	negative	negati ve	positive	positiv e	negative	negative	negative	negative	negative	negative
Mechanical ventilation	Yes	Yes	Yes	Yes	No	Yes	Yes	Yes	Yes	Yes
ARDS# classification	Severe	mode rate	Severe	Severe	Moderate	Severe	Severe	moderat e	Severe	Severe
History of contact	negative	positi ve	positive	negativ e	positive	negative	negative	positive	positive	negative
Died	Yes	No	Yes	No	No	Yes	Yes	No	No	Yes

*LOC: level of consciousness

#ARDS: acute respiratory distress syndrome

• UPJO: urethropelvic junction obstruction

∞ Immunodeficiency-10 is an autosomal recessive primary immunodeficiency characterized by the onset of recurrent infections in childhood due to defective T- and NK-cell function although the severity is variable (11).

Confidential: For Review Only

Table 2: Paraclinical findings on day one of admission

COVID Real-time PCR	positive						negative			
Patient	1	2	3	4	5	6	7	8	9	10
Ferritin (ng/ml) M: 22.81-275 F: 4.63-204	580	1235	954	5701	750	1310	3489	1067	651	*
Typical Chest CT finding	positive	positive	positive	positive	positive	#	positive	positive	positive	positive
White blood cells(count/ml)	26100	9800	7100	11300	7500	22000	14000	12000	3800	12000
Lymphocyte	2088	1470	710	1017	1100	1100	6380	2400	760	480
Procalcitonin ≤ 0.3	0.24	30.5	10.1	1.36	0.6	*	1.8	73	0.2	*
C- reactive protein <6 (mg/L)	32	90	50	150	37	5	3	85	3	3
Creatine phosphokinase (U/L) M: < 171 F:< 145	37	1769	950	374	557	264	88	220	163	140
Lactate dehydrogenase (U/L) < 480	640	635	5660	1820	1350	1350	1150	786	1670	938
Troponin (ng/ml) < 19	32	29.2	3343	2029	1.5	450	27	68	35	1.5
D-Dimer (ng/ml) < 500	1924	540	10000	9232	1296	638	5135	9536	1296	10000
Total bilirubin 0.1-1.2	0.7	0.3	35	0.6	0.4	0.2	*	0.3	0.6	*
Direct bilirubin < 0.3	0.3	0.1	21	0.1	0.2	0.1	*	0.1	0.1	*
Aspartate transaminase (U/L) M: < 37 F: < 31	1020	36	2030	51	40	97	*	77	68	*
Alanine aminotransferase (U/L) M: < 41 F: < 31	52	14	690	37	33	13	*	28	34	*
Creatinine M: 0.8 - 1.3 F: 0.6 - 1.2	0.9	0.5	0.1	0.5	0.9	4.8	0.5	0.4	0.4	3.3
Blood urea nitrogen (mg/dl) 8 - 20	13	11	113	13	14	53	9	3	19	62

M: male/F: female

was not performed due to his unstable condition

*did not checked

Discussion

Confidential: For Review Only

The reported overall case fatality rate is 2.3% according to China Centre for Disease Control and Prevention (CDC) which is much lower than SARS (9.6%) and MERS (34%), but significantly higher compared to the latest H1N1 influenza pandemic (0.001 – 0.007%) (12). However, WHO reported the global case fatality rate as high as 4.4% with absolute number of deaths already higher than the total fatality of SARS and MERS combined (13). The case fatality reported from Italy is 7.2%, which increased to 9.8% (WHO as on March 26, 2020) (14).

There are few studies regarding pediatric patients admitted in PICU The case fatality rate in our PICU was 50%. It was also about 50% in adult patients admitted in ICU (15,16,17) . However, pediatric data from Madrid, Spain , reported no mortality (18), and in a Chinese study there was only one case (19). In a cohort study in children hospital in Wuhan, three pediatric patients were admitted in the PICU, all with comorbidities and one case (33%) died (20). In another study from Wuhan, one patient without comorbidity was admitted in PICU and survived (5).

In our study, all patients were febrile and 70% had cough, but in some studies non-critical pediatric fever was present in less than 50%(5, 19, 20).

In our study, 60% of patients had positive RT-PCR FOR COVID-19. There are some reasons for that; we tested the patients via oropharyngeal swab only and nasopharyngeal swab was not done. The other possibility is that the patients arrived in the PICU in terminal stages of the disease, so the viral shedding was low at that time.

Moreover, in a study on 48 pediatric patients with COVID-19 admitted in 46 North American PICUs, 83% had significant pre-existing comorbidities, 73% presented with respiratory symptoms, 38% required invasive ventilation, and 23% had failure of 2 or more organ systems. The mortality rate was 4% in their study (up to the time of the report). Three patients were intubated, one patient was taking extracorporeal membrane oxygenation(ECMO), and only 25% needed vasoactive support (21).

In the beginning of the pandemic, it was assumed that the main organ involvement in COVID-19 was respiratory system, but several studies reported Kawasaki-like syndrome MIS-C later (22).

Confidential: For Review Only

DeBiasi et al. described a 177 pediatric patients series in the Washington, DC metropolitan region; among them, 9 cases required critical care, 8 needed respiratory support, and one had Kawasaki-like shock syndrome(23).

Another report mentioned 8 pediatric patients with hyperinflammatory shock, showing features similar to atypical Kawasaki disease, Kawasaki disease shock syndrome, or toxic shock syndrome, all of which had negative test for severe acute respiratory syndrome coronavirus 2 (SARS-CoV-2) on arrival, but clinical and laboratory findings were in favor of the disease. Two patients' tests for SARS-COV-2 was positive on discharge. Seven patients needed norepinephrine infusion, and one passed away according to their report (24).

In our study, some of our severely infected patients had elevated troponin level (especially in patients number 3,4,6), but despite fulfilling the criteria for MIS-C on arrival to PICU, they were hypotensive; this may explain those high levels, but could not rule out MIS-C.

Also, in the present study, six patients needed norepinephrine in addition to dopamine/epinephrine infusion due to refractory shock. In patient number 9, low amount of SVRI was the reason of norepinephrine infusion.

It has been shown that coronavirus infection (SARS, MERS and even; COVID-19) could damage the liver, and mild to moderate elevation of Alanine aminotransferase (ALT); decreased albumin and increased serum bilirubin levels occur frequently (25,26,27,28), but extremely high ALT and bilirubin levels in patient number 3 was noteworthy with some probable explanation like acute liver failure caused by COVID-19 or a rare complication of the disease (29).

In patient number 3, BUN and serum creatinine levels were disproportionate; it should be noted that the patient was hypotensive and oliguric, but in a day after PICU admission, as the patient's blood pressure became stable and the patient had normal urination, BUN decreased to 85 and creatinine turned to 0.5. This could be explained by hypotension,, altered liver function and hepatorenal syndrome.

This was our first experience toward COVID-19 patients. As the time passed, we became more familiar with characteristics of the disease, and the ways to better diagnose, and manage it. Through reporting the characteristics of our patients, we aimed to share our experience regarding COVID 19 patients and highlight some dark points of the disease. It is suggested that a more valid and strict

Confidential: For Review Only

triage criteria should be chosenfor pediatric emergency rooms and the physicians and nurses engaged in pediatric care should be educated to better diagnose COVID 19 cases in this age group.

Conclusion

There is a general conception that pediatric patients infected by COVID-19 have less severe symptoms and better outcomes, but severe and fatal cases occur as well.

Conflict of interest: none

Funding: The authors did not receive a specific grant for this research from any funding agency in the public, commercial or not-for-profit sectors.

Authors' Contributions: A.S. planned the study and wrote the manuscript. E.S. gathered patients' data and submitted the manuscript. A.S.D. did the data interpretation A.S.D. and M.R.K. edited the manuscript and were scientific consultant. All authors discussed the results and contributed to the final manuscript.

Acknowledgements: The authors would like to thank Shiraz University of Medical Sciences, Shiraz, Iran and also Center for Development of Clinical Research of Namazi Hospital and Dr. Nasrin Shokrpour for editorial assistance.

Confidential: For Review Only

References

1. Lu H, Stratton CW, Tang YW. Outbreak of pneumonia of unknown etiology in Wuhan China: the mystery and the miracle. J Med Virol 2020; 92:401-2.

2. Zhu N, Zhang D, WangW, et al; China Novel Coronavirus Investigating and Research Team. A novel coronavirus from patients with pneumonia in China, 2019 [published January 24, 2020]. N Engl JMed.doi:10.1056/NEJMoa2001017

3. World Health Organization. Situation Report 1 2020 (World Health Organization. Novel coronavirus (2019-nCoV), situation report-1. 21 January 2020 [Internet]. Geneva

4. Chan JF, Yuan S, Kok KH, To KK, Chu H, Yang J, et al. A familial cluster of pneumonia associated with the 2019 novel coronavirus indicating person-to-person transmission: a study of a family cluster. Lancet. 2020;395(10223):514-23.

5. Liu W, Zhang Q, Chen J, Xiang R, Song H, Shu S, et al. Detection of Covid-19 in Children in Early January 2020 in Wuhan, China. N Engl J Med. 2020.

6. Cao Q, Chen YC, Chen CL, Chiu CH. SARS-CoV-2 infection in children: Transmission dynamics and clinical characteristics. J Formos Med Assoc. 2020.

7. World Health Organization. (2020). Multisystem inflammatory syndrome in children and adolescents with COVID-19: scientific brief, 15 May 2020. World Health Organization. https://apps.who.int/iris/handle/10665/332095. License: CC BY-NC-SA 3.0 IGO

8. Chu DKW, Pan Y, Cheng SMS, et al. Molecular Diagnosis of a Novel Coronavirus (2019-nCoV) Causing an Outbreak of Pneumonia. Clin Chem. 2020;66(4):549-555. doi:10.1093/clinchem/hvaa029

9. Zhang, Zu-Li, Yu-Lei Hou, De-Tao Li, and Feng-Zeng Li. "Laboratory Findings of Covid-19: A Systematic Review and Meta-Analysis." Scandinavian Journal of Clinical and Laboratory Investigation (2020): 1-7. https://doi.org/10.1080/00365513.2020.1768587. https://doi.org/10.1080/00365513.2020.1768587.

Confidential: For Review Only

10. Foust, Alexandra M., Grace S. Phillips, Winnie C. Chu, Pedro Daltro, Karuna M. Das, Pilar Garcia-Peña, Tracy Kilborn, Abbey J. Winant, and Edward Y. Lee. "International Expert Consensus Statement on Chest Imaging in Pediatric Covid-19 Patient Management: Imaging Findings, Imaging Study Reporting and Imaging Study Recommendations." Radiology: Cardiothoracic Imaging 2, no. 2 (2020):e200214.https://doi.org/10.1148/ryct.2020200214.

11. Parry, D. A., Holmes, T. D., Gamper, N., El-Sayed, W., Hettiarachchi, N. T., Ahmed, M., Cook, G. P., Logan, C. V., Johnson, C. A., Joss, S., Peers, C., Prescott, K., Savic, S., Inglehearn, C. F., Mighell, A. J. A homozygous STIM1 mutation impairs store-operated calcium entry and natural killer cell effector function without clinical immunodeficiency. J. Allergy Clin. Immun. 137: 955-957, 2016.[PubMed: 26560041, images] [Full Text]

12. Wu Z, McGoogan JM. Characteristics of and important lessons from the coronavirus disease 2019 (COVID-19) outbreak in China. JAMA. Published online February 24, 2020. doi:10.1001/jama.2020.2648.

13. Mahase E. Coronavirus: covid-19 has killed more people than SARS and MERS combined, despite lower case fatality rate. BMJ. 2020; 368:m641.

14. Onder G, Rezza G, Brusaferro S. Case-fatality rate and characteristics of patients dying in relation to COVID-19 in Italy. JAMA. Published online March 23, 2020. doi:10.1001/jama.2020.4683.

15. Arentz M, Yim E, Klaff L, et al. Characteristics and outcomes of 21 critically ill patients with COVID-19 in Washington State. JAMA 2020 March 19 (Epub ahead of print).

16. Guan W, Ni Z, Hu Y, et al. Clinical characteristics of coronavirus disease 2019 in China. N Engl J Med. DOI: 10.1056/ NEJMoa2002032.

17. Bhatraju, P. K., et al. (2020). "Covid-19 in Critically Ill Patients in the Seattle Region — Case Series." New England Journal of Medicine.

18. Tagarro, A., et al. (2020). "Screening and Severity of Coronavirus Disease 2019 (COVID-19) in Children in Madrid, Spain." JAMA Pediatr.

19. Lu, X., et al. (2020). "SARS-CoV-2 Infection in Children." New England Journal of Medicine.

Confidential: For Review Only

20. Dong Y, Mo X, Hu Y, et al: Epidemiological characteristics of 2143 pediatric patients with 2019 coronavirus disease in China. Pediatrics 2020 Mar 16. [Online ahead of print]

21. Shekerdemian LS, Mahmood NR, Wolfe KK, et al. Characteristics and Outcomes of Children With Coronavirus Disease 2019 (COVID-19) Infection Admitted to US and Canadian Pediatric Intensive Care Units [published online ahead of print, 2020 May 11]. JAMA Pediatr. 2020;10.1001/jamapediatrics.2020.1948. doi:10.1001/jamapediatrics.2020.1948

22. Jones VG, Mills M, Suarez D, Hogan CA, Yeh D, Bradley Segal J, et al. COVID-19 and Kawasaki Disease: Novel Virus and Novel Case. Hosp Pediatr. 2020. Epub 2020/04/09

23.DeBiasi RL, Song X, Delaney M, Bell M, Smith K, Pershad J, et al. Severe COVID-19 in children and young adults in the Washington, DC metropolitan region. J Pediatr. 2020 May 13 [Epub ahead of print]. Available from: https://dx.doi.org/10.1016/j.jpeds.2020.05.007

24. Riphagen S, Gomez X, Gonzalez-Martinez C, Wilkinson N, Theocharis P. Hyperinflammatory shock in children during COVID-19 pandemic. Lancet. 2020;395(10237):1607-1608. doi:10.1016/S0140-6736(20)31094-1

25. Lu S-N, Lee C-M, Lin M-C, et al. Sequential changes of serum aminotransferase levels in patients with severe acute respiratory syndrome.

Am J Trop Med Hyg. 2004; 71(2):125-128.

26. Arabi YM, Al-Omari A, Mandourah Y, et al. Critically Ill patients with the Middle East respiratory syndrome. Crit Care Med. 2017; 45(10):1683-1695.

27. Xu, L, Liu, J, Lu, M, Yang, D, Zheng, X. Liver injury during highly pathogenic human coronavirus infections. LiverInt. 2020; 00: 1 7. https://doi.org/10.1111/liv.14435

28. Huang C, Wang Y, Li X, et al. Clinical features of patients infected with 2019 novel coronavirus in Wuhan, China. Lancet 2020; 395: 497–506.

29. Saeed A, Shorafa E, Shahramian I, Afshari M, Salahifard M , et al. An 11-Year-Old Boy Infected with COVID-19 with Presentation of Acute Liver Failure,

Confidential: For Review Only

Hepat Mon. Online ahead of Print ; In Press(In Press):e104415. doi: 10.5812/hepatmon.104415.

Confidential: For Review Only

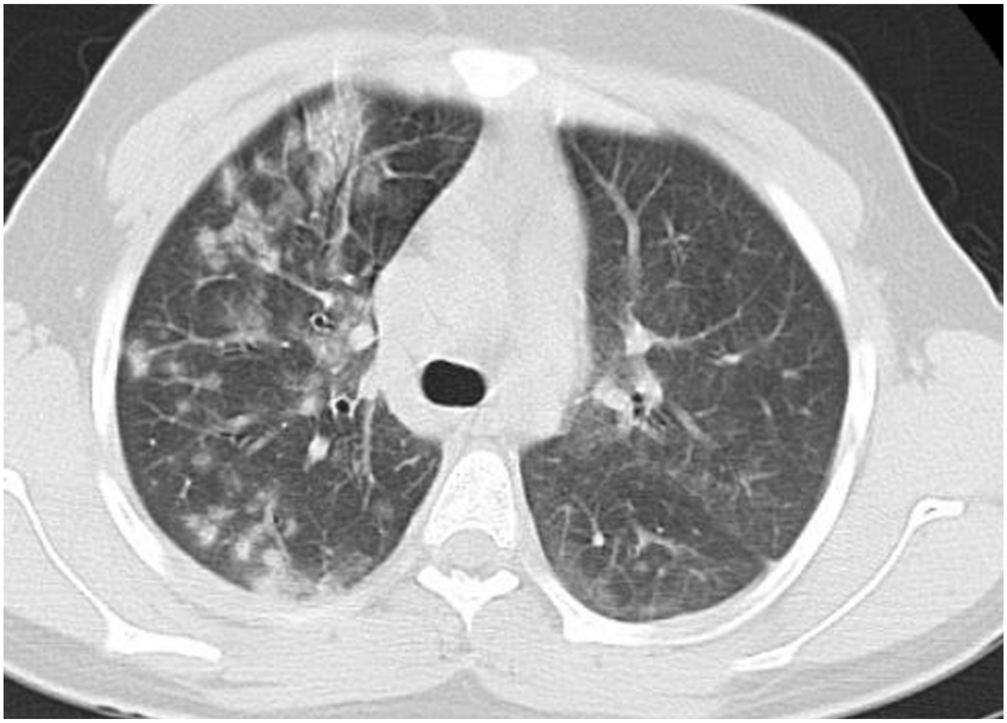

82x58mm (300 x 300 DPI)

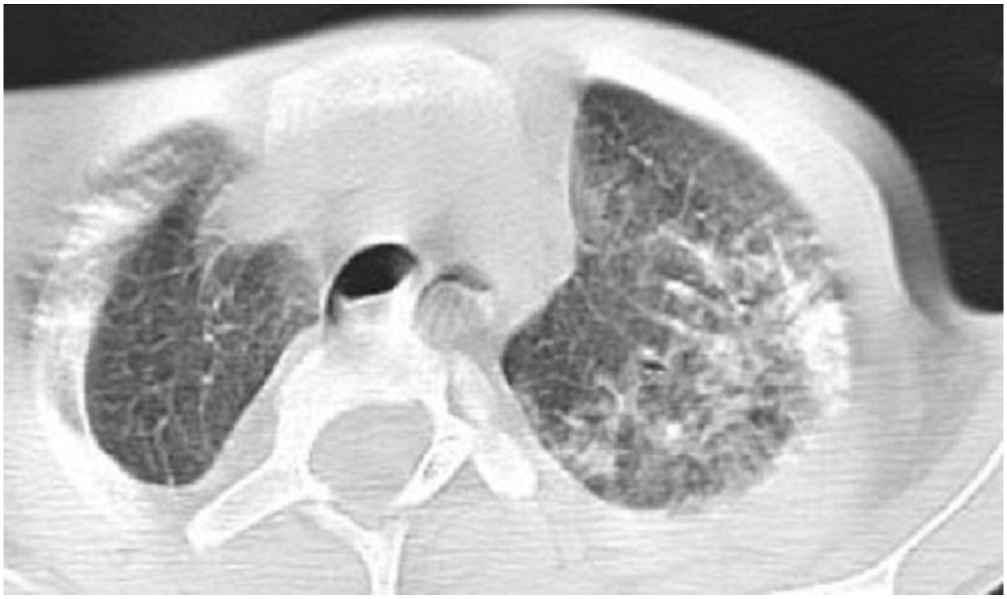

529x312mm (96 x 96 DPI)

BMJ Paediatrics Open

Clinical presentation of pediatric patients with COVID 19 admitted to a single paediatric intensive care unit (PICU) in Iran

Journal:	*BMJ Paediatrics Open*
Manuscript ID	bmjpo-2020-000715.R2
Article Type:	Original research
Date Submitted by the Author:	26-Aug-2020
Complete List of Authors:	Saeed, Amir; Shiraz University of Medical Sciences Medical School, Pediatrics<br>shorafa, eslam; Shiraz University of Medical Sciences,<br>sanaeidashti, anahita; Shiraz University of Medical Sciences, department of pediatrics<br>kadivar, mohammad rahim; Shiraz University of Medical Sciences, department of pediatrics
Keywords:	Health services research, Virology, Mortality

SCHOLARONE™
Manuscripts

I, the Submitting Author has the right to grant and does grant on behalf of all authors of the Work (as defined in the below author licence), an exclusive licence and/or a non-exclusive licence for contributions from authors who are: i) UK Crown employees; ii) where BMJ has agreed a CC-BY licence shall apply, and/or iii) in accordance with the terms applicable for US Federal Government officers or employees acting as part of their official duties; on a worldwide, perpetual, irrevocable, royalty-free basis to BMJ Publishing Group Ltd ("BMJ") its licensees and where the relevant Journal is co-owned by BMJ to the co-owners of the Journal, to publish the Work in this journal and any other BMJ products and to exploit all rights, as set out in our licence.

The Submitting Author accepts and understands that any supply made under these terms is made by BMJ to the Submitting Author unless you are acting as an employee on behalf of your employer or a postgraduate student of an affiliated institution which is paying any applicable article publishing charge ("APC") for Open Access articles. Where the Submitting Author wishes to make the Work available on an Open Access basis (and intends to pay the relevant APC), the terms of reuse of such Open Access shall be governed by a Creative Commons licence – details of these licences and which Creative Commons licence will apply to this Work are set out in our licence referred to above.

Other than as permitted in any relevant BMJ Author's Self Archiving Policies, I confirm this Work has not been accepted for publication elsewhere, is not being considered for publication elsewhere and does not duplicate material already published. I confirm all authors consent to publication of this Work and authorise the granting of this licence.

Confidential: For Review Only

Clinical presentation of pediatric patients with COVID 19 admitted to a single paediatric intensive care unit (PICU) in Iran

1. **Eslam Shorafa**

Assistant Professor of Pediatric Intensive Care, Department of Pediatrics, Division of Intensive Care Unit, Shiraz University of Medical Sciences, Shiraz, Iran.

E-mail; omidshorafa@gmail.com

Postal address: Khalili street, Namazi complex, apartment A, unit 3, Shiraz, Iran

Phone number; +989363946906, fax number: +9836474298

2. **Amir Saeed**

Assistant Professor of Pediatric Intensive Care, Department of Pediatrics, Division of Intensive Care Unit, Shiraz University of Medical Sciences, Shiraz, Iran.

E-mail; dr.saeedamir@yahoo.com

3. **Anahita Sanaei Dashti**

Professor of Pediatric Infectious Diseases, Professor Alborzi Clinical Microbiology Research Center, Shiraz University of Medical Sciences, Shiraz, Iran.

E-mail; anahitasanaei@gmail.com

4. **Mohammad Rahim Kadivar**

Professor of Pediatric Infectious Diseases, Professor Alborzi Clinical Microbiology Research Center, Shiraz University of Medical Sciences, Shiraz, Iran.

E-mail; Kadmr1@gmail.com

Word count: 1522

Keywords: COVID 19; SARS coronavirus 2 , Wegener , child, PICU

Confidential: For Review Only

Abstract

Objectives: To describe the clinical characteristics of pediatric patients admitted to a single pediatric intensive care unit (PICU) in Iran with COVID-19

Methods: A cross- sectional study of pediatric patients who were admitted to a COVID dedicated PICU from March 16 to April 21, 2020 with COVID-19.

Results: Six children had confirmed COVID-19 and four had suspected COVID-19. Six had pre-existing chronic medical conditions. Nine had respiratory failure and needed ventilation. Five children, of whom four had chronic medical conditions, died. Four had cardiac arrhythmias. Clinical presentation included fever and cough.

Conclusion: COVID-19 can be fatal in pediatric patients, especially in those with a chronic medical condition.

What is known about the subject?

► COVID -19 infection is less frequent and severe in pediatric patients than adult patients.

► The main symptom is fever and few pediatric patients need mechanical ventilation or inotrope support.

What this study adds?

► COVID-19 infection may be fatal in pediatric patients, especially in those with chronic medical conditions.

►The main respiratory problems in severe cases are oxygenation failure and a hypotensive state necessitating inotropes.

► The main causes of mortality in our study were cardiac arrhythmia and refractory hypoxemia.

Confidential: For Review Only

Confidential: For Review Only

Introduction

In December 2019, a cluster of acute respiratory illness occurred in Wuhan, Hubei Province, China by a novel coronavirus named severe acute respiratory syndrome coronavirus-2 (SARS-CoV-2) (1). On the 3rd of January 2020, the virus was identified in the samples of bronchoalveolar lavage fluid from a patient in Wuhan and was confirmed as the cause of the disease (2).

The current outbreak of infections with SARS-CoV-2 was termed Coronavirus Disease 2019 (COVID-19) by the World Health Organization (WHO) (3). The disease rapidly spread from Wuhan to other areas of the world, so that the world health organization (WHO) announced Covid-19 outbreak as a pandemic on March 11, 2020 (4).

The 2019-nCoV is closely similar to bat coronaviruses, and it has been assumed that bats are the primary source. While the origin of the 2019-nCoV is still being investigated, the current evidence suggests that spread to humans occurred from wild animals illegally sold in the Huanan Seafood Wholesale Market.

The rate of infection with covid-19 in children is low and the first pediatric case was reported on January 20, 2020, who was a 10-year-old Chinese boy, whose family had visited Wuhan City (4). A retrospective study on 366 children aged under 16 years old and hospitalized for respiratory infections between January 7 and 15, 2020, confirmed COVID-19 infection in six (1.6%) of them. This study suggests that COVID-19 infections in children occurred early in the epidemic (5). One reason for the low frequency of the disease in children in China could

be the fact that children were rarely tested for the virus in the earlier phase of the outbreak, especially in Hubei province, where most of the patients were confirmed, so there are a limited data on the prevalence of COVID-19 in children (6). The virus can produce a Kawasaki-like illness in children. World health organization (WHO) developed a preliminary case definition for this condition which was later named ‘multisystem inflammatory disorder’ in COVID-19 (MIS-C). The syndrome should be considered in children with features of typical or atypical Kawasaki disease or toxic shock syndrome (7).

Herein, we describe the patients with COVID-19 presenting with multi-system inflammatory syndrome. In Iran, the first COVID-19 cases were detected in February 2020 in Qom city. There are a few reports from critically ill pediatric patients in the country. In this article, we describe the characteristics of pediatric patients with COVID-19 admitted in pediatric intensive care unit (PICU) of Namazi hospital in Shiraz, Iran.

Methods

Nemazi hospital in Shiraz, is the largest and the main tertiary referral center in the south of Iran with more than one thousand beds. The medical PICU of this hospital has 18 beds with two separate sections, one of which was devoted to COVID 19 cases from the beginning of the outbreak and those with suspected or confirmed COVID-19 were admitted in this ward. All children aged one month to 18 years admitted to the COVID-19 PICU between March 16 and 21 April were included.

A confirmed case of Covid-19 was defined by a positive result on a reverse transcriptase–polymerase chain-reaction (RT-PCR) assay of a specimen collected on an oropharyngeal swab, nasopharyngeal swab or bronchoalveolar lavage. The tests were performed using an Applied Biosystem Step One plus real-time PCR machine (Applied Biosystem, CA, USA). Amplification of N and ORF1b took place in a 20μL single-tube, and Superscript III Platinum one-step quantitative RT-PCR system (Invitrogen, Carlsbad, CA). Reactions contained 10.0 μL of 2X RT/PCR reaction mix, 1 μL primers/prob mix, 0.4 μL Superscript III RT/Platinum Taq mix, 0.4 μL ROX reference dye, and 5 μL of extracted sample RNA or serially diluted previously confirmed patients’ positive control. The cycling conditions consisted of one cycle at 50°C for 10 min, one cycle at 95°C for 2 min, 45 cycles at 95°C for 5 s, and 60°C for 30 min)(8).

Confidential: For Review Only

A suspected case of COVID 19 was defined as a patient with clinical and laboratory findings (9) (table 1) plus chest CT findings consistent with COVID 19 infection (10)(figure 1,2) and history of close contact, but negative PCR result.

Table 1: laboratory and clinical findings in favor of COVID19 infection

Laboratory findings		clinical manifestation
increased	decreased	
CRP D.dimer<br>ESR ferritin<br>AST,ALT<br>procalcitonin<br>LDH	Albumin<br>lymphocytes	fever, cough, sore throat, headache, shortness of breath, nausea, vomiting, abdominal pain
		Sepsis and organ failure

Patient involvement

Patients were not directly involved in the design of this study.

Ethics statement

This study was approved by the ethics committee of Shiraz University of Medical Sciences (IR.sums.med.rec.1399.40). Written informed consent was obtained from the parents and sent to the ethics committee.

Results

From March 16 to April 21, 2020, six patients were admitted with confirmed COVID-19 and four had suspected COVID-19 (Tables 2,3). Only one of them was transferred from the Emergency Department (ER) with suspected COVID-19. Nine were transferred from other wards with other suspected diagnoses. The median age was 7.9 years. Eight patients were male. Six patients had chronic medical conditions (Table 2). The other four were previously healthy.

Five patients had a positive history of contact to confirmed or suspected cases (3 patients had contact to positive RT-PCR cases) or possible cases with fever and cough. The main symptoms were fever, cough, abdominal pain, lethargy and encephalopathy. The median time from the presentation of symptoms to PICU admission was 4.7 days (range 3-8 days).

Confidential: For Review Only

All the children had tachypnoea and tachycardia on admission to the PICU. All patients had Pa02/Fio2 less than 300 and nine patients needed intubation and mechanical ventilation (all were intubated due to respiratory failure outside the PICU). One patient responded to oxygen supplementation via non-rebreathing mask despite decreased o2 saturation and PaO2 of 47 mmHg.

In other intubated patients, the main problem was severe hypoxia. Although they needed a low peak inspiratory pressure (PIP) for acceptable tidal volume, their oxygen saturation could not reach 85% , so peak expiratory pressure (PEEP) was increased even up to 18 and prone positioning was ordered to increase the oxygen saturation. (cases number 4 and 6). Extracorporeal membrane oxygenation (ECMO) was not available in our center.

Unfortunately, five patients died. Four of the five had chronic medical conditions. Cardiac arrhythmias occurred in three of the children who died.

Nine/Ten children had hypotension. Six children received high dose of norepinephrine(more than 0.3 micro/kilogram/minutes) in addition to other inotropes. Enoxaparin was started with the aim of antithrombotic prophylaxis in all the patients. In all patients broad spectrum antibiotics (meropenem and vancomycin) were started in addition to hydroxychloroquine (EKG was taken first and all had normal QTC; equal to or less than 0.40s) and kaletra (lopinavir/ritonavir), and intravenous immune globulin was given (IVIG) to 8 patients due to severe septic shock (except for cases number 5 and 10). In hypotensive patients, hydrocortisone, ascorbic acid, and thiamine were started.

In all of the patients, blood culture, tracheal aspirate, urine culture, and PCR for influenza A and B were negative (Table 2,3).

Patient number 3 presented with fever, abdominal pain and tachypnea. he was transferred by his parents to our center with the diagnosis of acute liver failure (Nemazi hospital is the referral center for liver transplant in Iran). On arrival, he was intubated due to decreased o2 saturation and decreased level of consciousness. He had hypotension, fever and patchy ground glass infiltrations in the chest CT scan. Surprisingly, the serum bilirubin level was very high (total bilirubin was 35); the other lab data in addition to alkaline phosphatase:387 and Gamma-glutamyl transferase (GGT) was:45 are shown in Table 3.The patient tests for hepatitis A, B, C, EBV, HSV and CMV were negative and serum

Confidential: For Review Only

ceruloplasmin level and LKM Ab were in normal range; he had a negative history of taking medications or substance abuse. His parents had fever and cough and their nasopharyngeal RT-PCR for COVID 19 turned positive in both.

Table 2: clinical details of the patients

Patient number	1	2	3	4	5	6	7	8	9	10
Real-time PCR	+	+	+	+	+	+	-	-	-	-
Age(years)	4 months	3	11	13	15	16	1	1.5	3	16
Sex	male	male	male	male	male	male	male	female	female	male
Hypotension	Yes	Yes	Yes	Yes	No	Yes	Yes	Yes	Yes	Yes
Tachycardia on admission	Yes	Yes	Yes	Yes	Yes	Yes	Yes	Yes	Yes	Yes
Tachypnea on admission	Yes	Yes	Yes	Yes	Yes	Yes	Yes	Yes	Yes	Yes
Chief complaint(s)	Fever & Letharginess	Fever & cough	Fever & abdominal pain & decreased LOC	Fever & Letharginess	Fever & cough	Fever & cough	Fever & Letharginess	Fever & cough	Fever & cough	Fever & cough
Previous illness	Cerebral Shunt	negative	negative	Cerebral palsy	negative	wegener granolomatosis	UPJO• with nephrostomy	negative	Immunodeficiency∞	Cerebral palsy
Convulsion	Yes	No	Yes	No	No	No	No	Yes	Yes	No
Decreased LOC*	negative	negative	positive	positive	negative	negative	negative	negative	negative	negative
Mechanical ventilation	Yes	Yes	Yes	Yes	No	Yes	Yes	Yes	Yes	Yes
ARDS# classification	Severe	moderate	Severe	Severe	Moderate	Severe	Severe	moderate	Severe	Severe
History of contact	negative	positive	positive	negative	positive	negative	negative	positive	positive	negative
Died	Yes	No	Yes	No	No	Yes	Yes	No	No	Yes

*LOC: level of consciousness

#ARDS: acute respiratory distress syndrome

• UPJO: urethropelvic junction obstruction

∞ Immunodeficiency-10 is an autosomal recessive primary immunodeficiency characterized by the onset of recurrent infections in childhood due to defective T- and NK-cell function although the severity is variable (11).

Confidential: For Review Only

Table 3: laboratory results on day one of admission

COVID Real-time PCR	positive						negative			
Patient	1	2	3	4	5	6	7	8	9	10
Ferritin (ng/ml) M: 22.81-275 F: 4.63-204	580	1235	954	5701	750	1310	3489	1067	651	*
Typical Chest CT finding	positive	positive	positive	positive	positive	#	positive	positive	positive	positive
White blood cells(count/ml)	26100	9800	7100	11300	7500	22000	14000	12000	3800	12000
Lymphocyte	2088	1470	710	1017	1100	1100	6380	2400	760	480
Procalcitonin ≤ 0.3	0.24	30.5	10.1	1.36	0.6	*	1.8	73	0.2	*
C- reactive protein <6 (mg/L)	32	90	50	150	37	5	3	85	3	3
Creatine phosphokinase (U/L) M: < 171 F:< 145	37	1769	950	374	557	264	88	220	163	140
Lactate dehydrogenase (U/L) < 480	640	635	5660	1820	1350	1350	1150	786	1670	938
Troponin (ng/ml) < 19	32	29.2	3343	2029	1.5	450	27	68	35	1.5
D-Dimer (ng/ml) < 500	1924	540	10000	9232	1296	638	5135	9536	1296	10000
Total bilirubin 0.1-1.2	0.7	0.3	35	0.6	0.4	0.2	*	0.3	0.6	*
Direct bilirubin < 0.3	0.3	0.1	21	0.1	0.2	0.1	*	0.1	0.1	*
Aspartate transaminase (U/L) M: < 37 F: < 31	1020	36	2030	51	40	97	*	77	68	*
Alanine aminotransferase (U/L) M: < 41 F: < 31	52	14	690	37	33	13	*	28	34	*
Creatinine M: 0.8 - 1.3 F: 0.6 - 1.2	0.9	0.5	0.1	0.5	0.9	4.8	0.5	0.4	0.4	3.3
Blood urea nitrogen (mg/dl) 8 - 20	13	11	113	13	14	53	9	3	19	62

M: male/F: female

was not performed due to his unstable condition

*did not checked

Confidential: For Review Only

Discussion

There are few studies regarding pediatric patients admitted in PICU. Pediatric data from Madrid, Spain , reported no mortality, but described one child who needed mechanical ventilation and two who needed non-invasive ventilation(12). In a Chinese study there was only one case who needed mechanical ventilation (13). In a cohort study in children hospital in Wuhan, three pediatric patients were admitted in the PICU, all with comorbidities and one case died (14). In another study from Wuhan, one patient without comorbidity was admitted in PICU and survived (5).

In our study, all patients were febrile and 70% had cough, but in some studies non-critical pediatric fever was present in less than 50%(5, 13, 14).

In a study on 48 pediatric patients with COVID-19 admitted in 46 North American PICUs, 83% had significant pre-existing comorbidities, 73% presented with respiratory symptoms, 38% required invasive ventilation, and 23% had failure of 2 or more organ systems. The mortality rate was 4% in their study (up to the time of the report). Three patients were intubated, one patient was taking extracorporeal membrane oxygenation(ECMO), and only 25% needed vasoactive support (15).

In the beginning of the pandemic, it was assumed that the main organ involvement in COVID-19 was respiratory, but several studies have reported Kawasaki- like syndrome MIS-C later (16).

DeBiasi et al. described a 177 pediatric patients series in the Washington, DC metropolitan region; among them, 9 cases required critical care, 8 needed respiratory support, and one had Kawasaki-like shock syndrome (17).

In our study, some of our severely infected patients had elevated troponin level and fulfilling the criteria for MIS-C on arrival to PICU. Also hypotension and organ hypo perfusion, can explain the etiology of high level of troponin.

It has been shown that coronavirus infection (SARS, MERS and even; COVID-19) could damage the liver, and mild to moderate elevation of Alanine aminotransferase (ALT); decreased albumin and increased serum bilirubin levels occur frequently (18,19,20,21), but extremely high ALT and bilirubin levels in patient number 3 was noteworthy with some probable explanation like acute liver failure caused by COVID-19 or a rare complication of the disease (22).

Confidential: For Review Only

Through reporting the characteristics of our patients, we aim to share our experience regarding COVID 19 patients.

There is a general conception that pediatric patients infected by COVID-19 have less severe symptoms and better outcomes, but severe and fatal cases occur as well.

Conflict of interest: none

Funding: The authors did not receive a specific grant for this research from any funding agency in the public, commercial or not-for-profit sectors.

Authors' Contributions: A.S. planned the study and wrote the manuscript. E.S. gathered patients' data and submitted the manuscript. A.S.D. did the data interpretation A.S.D. and M.R.K. edited the manuscript and were scientific consultant. All authors discussed the results and contributed to the final manuscript.

Acknowledgements: The authors would like to thank Shiraz University of Medical Sciences, Shiraz, Iran and also Center for Development of Clinical Research of Namazi Hospital and Dr. Nasrin Shokrpour for editorial assistance.

Confidential: For Review Only

References

1. Lu H, Stratton CW, Tang YW. Outbreak of pneumonia of unknown etiology in Wuhan China: the mystery and the miracle. J Med Virol 2020; 92:401-2.

2. Zhu N, Zhang D, WangW, et al; China Novel Coronavirus Investigating and Research Team. A novel coronavirus from patients with pneumonia in China, 2019 [published January 24, 2020]. N Engl JMed.doi:10.1056/NEJMoa2001017

3. World Health Organization. Situation Report 1 2020 (World Health Organization. Novel coronavirus (2019-nCoV), situation report-1. 21 January 2020 [Internet]. Geneva

4. Chan JF, Yuan S, Kok KH, To KK, Chu H, Yang J, et al. A familial cluster of pneumonia associated with the 2019 novel coronavirus indicating person-to-person transmission: a study of a family cluster. Lancet. 2020;395(10223):514-23.

5. Liu W, Zhang Q, Chen J, Xiang R, Song H, Shu S, et al. Detection of Covid-19 in Children in Early January 2020 in Wuhan, China. N Engl J Med. 2020.

6. Cao Q, Chen YC, Chen CL, Chiu CH. SARS-CoV-2 infection in children: Transmission dynamics and clinical characteristics. J Formos Med Assoc. 2020.

7. World Health Organization. (2020). Multisystem inflammatory syndrome in children and adolescents with COVID-19: scientific brief, 15 May 2020. World Health Organization. https://apps.who.int/iris/handle/10665/332095. License: CC BY-NC-SA 3.0 IGO

8. Chu DKW, Pan Y, Cheng SMS, et al. Molecular Diagnosis of a Novel Coronavirus (2019-nCoV) Causing an Outbreak of Pneumonia. Clin Chem. 2020;66(4):549-555. doi:10.1093/clinchem/hvaa029

9. Zhang, Zu-Li, Yu-Lei Hou, De-Tao Li, and Feng-Zeng Li. "Laboratory Findings of Covid-19: A Systematic Review and Meta-Analysis." Scandinavian Journal of Clinical and Laboratory Investigation (2020): 1-7. https://doi.org/10.1080/00365513.2020.1768587.
https://doi.org/10.1080/00365513.2020.1768587.

Confidential: For Review Only

10. Foust, Alexandra M., Grace S. Phillips, Winnie C. Chu, Pedro Daltro, Karuna M. Das, Pilar Garcia-Peña, Tracy Kilborn, Abbey J. Winant, and Edward Y. Lee. "International Expert Consensus Statement on Chest Imaging in Pediatric Covid-19 Patient Management: Imaging Findings, Imaging Study Reporting and Imaging Study Recommendations." Radiology: Cardiothoracic Imaging 2, no. 2 (2020):e200214.https://doi.org/10.1148/ryct.2020200214.

11. Parry, D. A., Holmes, T. D., Gamper, N., El-Sayed, W., Hettiarachchi, N. T., Ahmed, M., Cook, G. P., Logan, C. V., Johnson, C. A., Joss, S., Peers, C., Prescott, K., Savic, S., Inglehearn, C. F., Mighell, A. J. A homozygous STIM1 mutation impairs store-operated calcium entry and natural killer cell effector function without clinical immunodeficiency. J. Allergy Clin. Immun. 137: 955-957, 2016.[PubMed: 26560041, images] [Full Text]

12. Tagarro, A., et al. (2020). "Screening and Severity of Coronavirus Disease 2019 (COVID-19) in Children in Madrid, Spain." JAMA Pediatr.

13. Lu, X., et al. (2020). "SARS-CoV-2 Infection in Children." New England Journal of Medicine.

14. Dong Y, Mo X, Hu Y, et al: Epidemiological characteristics of 2143 pediatric patients with 2019 coronavirus disease in China. Pediatrics 2020 Mar 16. [Online ahead of print]

15. Shekerdemian LS, Mahmood NR, Wolfe KK, et al. Characteristics and Outcomes of Children With Coronavirus Disease 2019 (COVID-19) Infection Admitted to US and Canadian Pediatric Intensive Care Units [published online ahead of print, 2020 May 11]. JAMA Pediatr. 2020;10.1001/jamapediatrics.2020.1948. doi:10.1001/jamapediatrics.2020.1948

16. Jones VG, Mills M, Suarez D, Hogan CA, Yeh D, Bradley Segal J, et al. COVID-19 and Kawasaki Disease: Novel Virus and Novel Case. Hosp Pediatr. 2020. Epub 2020/04/09

17.DeBiasi RL, Song X, Delaney M, Bell M, Smith K, Pershad J, et al. Severe COVID-19 in children and young adults in the Washington, DC metropolitan region. J Pediatr. 2020 May 13 [Epub ahead of print]. Available from: https://dx.doi.org/10.1016/j.jpeds.2020.05.007

18. Lu S-N, Lee C-M, Lin M-C, et al. Sequential changes of serum aminotransferase levels in patients with severe acute respiratory syndrome.

Confidential: For Review Only

Am J Trop Med Hyg. 2004; 71(2):125-128.

19. Arabi YM, Al-Omari A, Mandourah Y, et al. Critically Ill patients with the Middle East respiratory syndrome. Crit Care Med. 2017; 45(10):1683-1695.

20. Xu, L, Liu, J, Lu, M, Yang, D, Zheng, X. Liver injury during highly pathogenic human coronavirus infections. LiverInt. 2020; 00: 1 7. https://doi.org/10.1111/liv.14435

21. Huang C, Wang Y, Li X, et al. Clinical features of patients infected with 2019 novel coronavirus in Wuhan, China. Lancet 2020; 395: 497–506.

22. Saeed A, Shorafa E, Shahramian I, Afshari M, Salahifard M , et al. An 11-Year-Old Boy Infected with COVID-19 with Presentation of Acute Liver Failure, Hepat Mon. Online ahead of Print ; In Press(In Press):e104415. doi: 10.5812/hepatmon.104415.

Figure legends:

Figure 1: chest CT of patient number 5

Figure 2: chest CT of patient number 10

Confidential: For Review Only

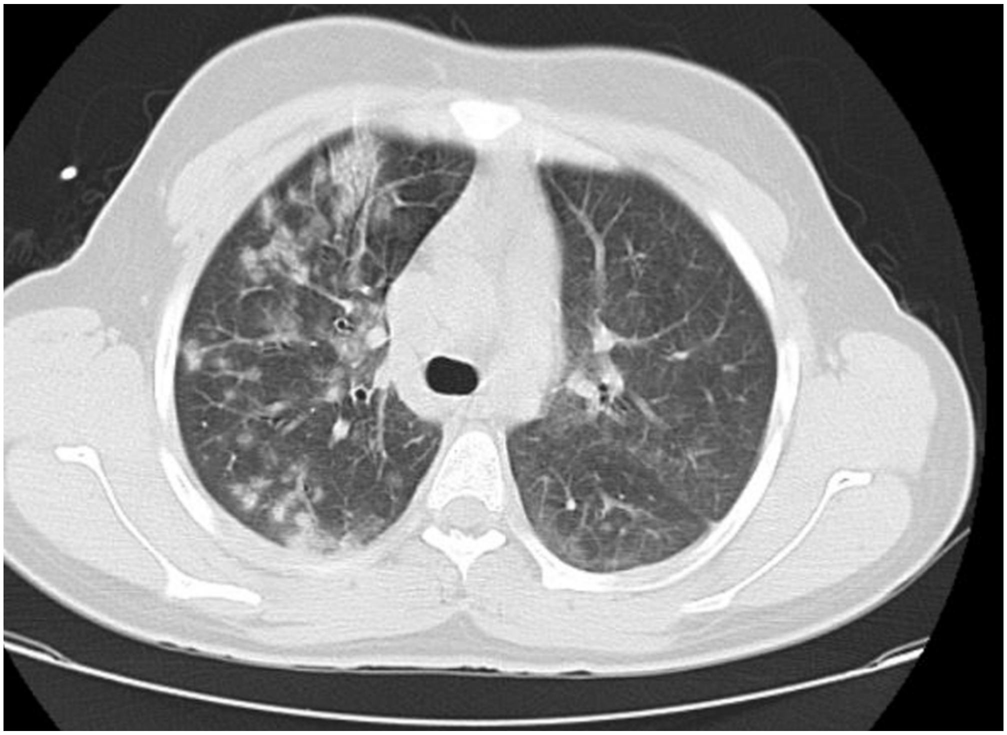

Figure 1: chest CT of patient number 5

99x72mm (300 x 300 DPI)

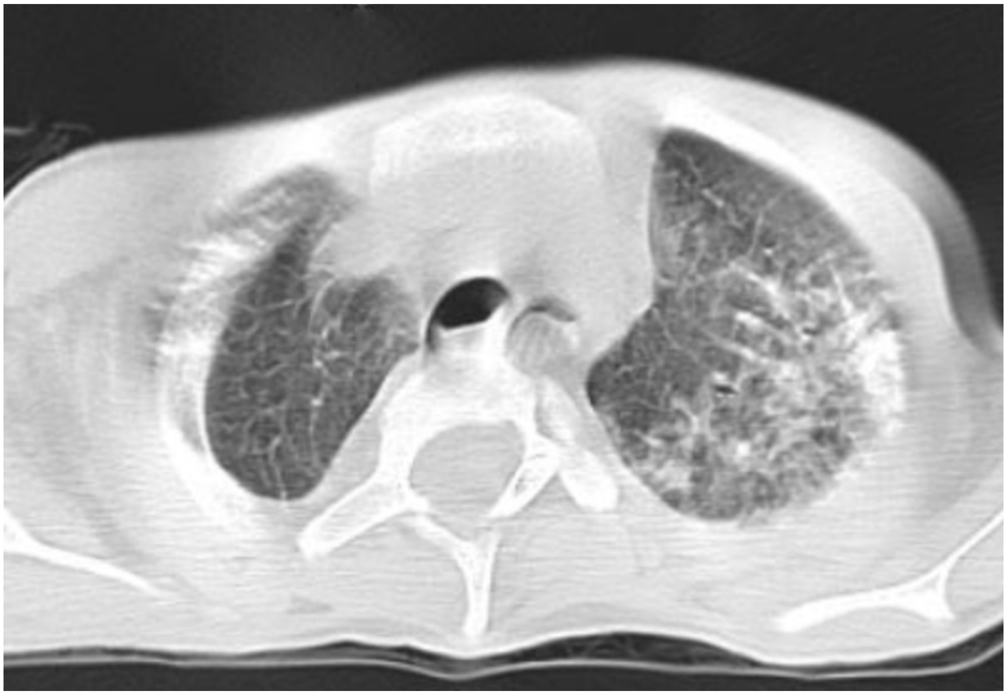

Figure 2: chest CT of patient number 10

99x68mm (300 x 300 DPI)

BMJ Paediatrics Open

Clinical presentation of pediatric patients with COVID 19 admitted to a single paediatric intensive care unit (PICU) in Iran

Journal:	*BMJ Paediatrics Open*
Manuscript ID	bmjpo-2020-000715.R3
Article Type:	Original research
Date Submitted by the Author:	29-Aug-2020
Complete List of Authors:	Saeed, Amir; Shiraz University of Medical Sciences Medical School, Pediatrics<br>shorafa, eslam; Shiraz University of Medical Sciences,<br>sanaeidashti, anahita; Shiraz University of Medical Sciences, department of pediatrics<br>kadivar, mohammad rahim; Shiraz University of Medical Sciences, department of pediatrics
Keywords:	Health services research, Virology, Mortality

SCHOLARONE™
Manuscripts

I, the Submitting Author has the right to grant and does grant on behalf of all authors of the Work (as defined in the below author licence), an exclusive licence and/or a non-exclusive licence for contributions from authors who are: i) UK Crown employees; ii) where BMJ has agreed a CC-BY licence shall apply, and/or iii) in accordance with the terms applicable for US Federal Government officers or employees acting as part of their official duties; on a worldwide, perpetual, irrevocable, royalty-free basis to BMJ Publishing Group Ltd ("BMJ") its licensees and where the relevant Journal is co-owned by BMJ to the co-owners of the Journal, to publish the Work in this journal and any other BMJ products and to exploit all rights, as set out in our licence.

The Submitting Author accepts and understands that any supply made under these terms is made by BMJ to the Submitting Author unless you are acting as an employee on behalf of your employer or a postgraduate student of an affiliated institution which is paying any applicable article publishing charge ("APC") for Open Access articles. Where the Submitting Author wishes to make the Work available on an Open Access basis (and intends to pay the relevant APC), the terms of reuse of such Open Access shall be governed by a Creative Commons licence – details of these licences and which Creative Commons licence will apply to this Work are set out in our licence referred to above.

Other than as permitted in any relevant BMJ Author's Self Archiving Policies, I confirm this Work has not been accepted for publication elsewhere, is not being considered for publication elsewhere and does not duplicate material already published. I confirm all authors consent to publication of this Work and authorise the granting of this licence.

Confidential: For Review Only

Clinical presentation of pediatric patients with COVID 19 admitted to a single paediatric intensive care unit (PICU) in Iran

1 . **Amir Saeed**

Assistant Professor of Pediatric Intensive Care, Department of Pediatrics, Division of Intensive Care Unit, Shiraz University of Medical Sciences, Shiraz, Iran.

E-mail; dr.saeedamir@yahoo.com

2. **Eslam Shorafa (corresponding author)**

Assistant Professor of Pediatric Intensive Care, Department of Pediatrics, Division of Intensive Care Unit, Shiraz University of Medical Sciences, Shiraz, Iran.

E-mail; omidshorafa@gmail.com

Postal address: Khalili street, Namazi complex, apartment A, unit 3, Shiraz, Iran

Phone number; +989363946906, fax number: +9836474298

3. **Anahita Sanaei Dashti**

Professor of Pediatric Infectious Diseases, Professor Alborzi Clinical Microbiology Research Center, Shiraz University of Medical Sciences, Shiraz, Iran.

E-mail; anahitasanaei@gmail.com

4. **Mohammad Rahim Kadivar**

Professor of Pediatric Infectious Diseases, Professor Alborzi Clinical Microbiology Research Center, Shiraz University of Medical Sciences, Shiraz, Iran.

E-mail; Kadmr1@gmail.com

Word count: 1522

Keywords: COVID 19; SARS coronavirus 2 , Wegener , child, PICU

Confidential: For Review Only

Abstract

Objectives: To describe the clinical characteristics of pediatric patients admitted to a single pediatric intensive care unit (PICU) in Iran with COVID-19

Methods: A cross- sectional study of pediatric patients who were admitted to a COVID dedicated PICU from March 16 to April 21, 2020 with COVID-19.

Results: Six children had confirmed COVID-19 and four had suspected COVID-19. Six had pre-existing chronic medical conditions. Nine had respiratory failure and needed ventilation. Five children, of whom four had chronic medical conditions, died. Four had cardiac arrhythmias. Clinical presentation included fever and cough.

Conclusion: COVID-19 can be fatal in pediatric patients, especially in those with a chronic medical condition.

What is known about the subject?

► COVID -19 infection is less frequent and severe in pediatric patients than adult patients.

► The main symptom is fever and few pediatric patients need mechanical ventilation or inotrope support.

What this study adds?

► COVID-19 infection may be fatal in pediatric patients, especially in those with chronic medical conditions.

►The main respiratory problems in severe cases are oxygenation failure and a hypotensive state necessitating inotropes.

► The main causes of mortality in our study were cardiac arrhythmia and refractory hypoxemia.

Confidential: For Review Only

Confidential: For Review Only

Introduction

The coronavirus pandemic originated in Wuhan, China[1 2] .The current outbreak of infections with coronavirus 2 (SARS-CoV-2) was termed Coronavirus Disease 2019 (COVID-19) by the World Health Organization (WHO)[3]. The disease rapidly spread from Wuhan to other areas of the world, so that the WHO announced that the outbreak was a pandemic in March 2020[4] .

The first paediatric case was a 10-year-old Chinese boy, whose family had visited Wuhan City[4] . A retrospective study on 366 children hospitalized for respiratory infections in January 2020 confirmed COVID-19 infection in six (1.6%) of them. This study suggests that COVID-19 infections in children occurred early in the epidemic[5] . Children were rarely tested for the virus in the earlier phase of the outbreak, so there are limited data on the prevalence of COVID-19 in children[6] . The virus can produce a Kawasaki-like illness in children. WHO developed a preliminary case definition for this condition which was later named 'multisystem inflammatory disorder' in COVID-19 (MIS-C)[7] .

In Iran, the first COVID-19 cases were detected in February 2020 in Qom city. There are a few reports from critically ill pediatric patients in the country. In this article, we describe the characteristics of pediatric patients with COVID-19 admitted in pediatric intensive care unit (PICU) of Namazi hospital in Shiraz, Iran.

Methods

Namazi hospital in Shiraz, is the largest and the main tertiary referral center in the south of Iran with more than one thousand beds. The medical PICU of this hospital has 18 beds with two separate sections, one of which was devoted to COVID 19 cases from the beginning of the outbreak and those with suspected or confirmed COVID-19 were admitted in this ward. All children aged one month to 18 years admitted to the COVID-19 PICU between March 16 and 21 April were included.

A confirmed case of Covid-19 was defined by a positive result on a reverse transcriptase–polymerase chain-reaction (RT-PCR) assay of a specimen collected on an oropharyngeal swab, nasopharyngeal swab or bronchoalveolar lavage. The tests were performed using an Applied Biosystem Step One plus real-time PCR machine (Applied Biosystem, CA, USA). Amplification of N and ORF1b took place in a 20µL single-tube, and Superscript III Platinum one-step quantitative RT-PCR system (Invitrogen, Carlsbad, CA). Reactions contained 10.0 µL of 2X RT/PCR reaction mix, 1 µL primers/prob mix, 0.4 µL Superscript III RT/Platinum Taq mix, 0.4 µL ROX reference dye, and 5 µL of extracted sample RNA or serially diluted previously confirmed patients' positive control. The cycling conditions consisted of one cycle at 50°C for 10 min, one cycle at 95°C for 2 min, 45 cycles at 95°C for 5 s, and 60°C for 30 min)[8].

A suspected case of COVID 19 was defined as a patient with clinical and laboratory findings[9] (table 1) plus chest CT findings consistent with COVID 19 infection[10] (figure 1,2) and history of close contact, but negative PCR result.

Table 1: laboratory and clinical findings in favor of COVID19 infection

Laboratory findings			clinical manifestation
increased		decreased	
CRP<br>ESR<br>AST,ALT<br>procalcitonin<br>LDH	D.dimer<br>ferritin	Albumin<br>lymphocytes	fever, cough, sore throat, headache, shortness of breath, nausea, vomiting, abdominal pain
			Sepsis and organ failure

Patient involvement

Patients were not directly involved in the design of this study.

Confidential: For Review Only

Ethics statement

This study was approved by the ethics committee of Shiraz University of Medical Sciences (IR.sums.med.rec.1399.40). Written informed consent was obtained from the parents and sent to the ethics committee.

Results

From March 16 to April 21, 2020, six patients were admitted with confirmed COVID-19 and four had suspected COVID-19 (Tables 2,3). Only one of them was transferred from the Emergency Department (ER) with suspected COVID-19. Nine were transferred from other wards with other suspected diagnoses. The median age was 7.9 years. Eight patients were male. Six patients had chronic medical conditions (Table 2). The other four were previously healthy.

Five patients had a positive history of contact to confirmed or suspected cases (3 patients had contact to positive RT-PCR cases) or possible cases with fever and cough. The main symptoms were fever, cough, abdominal pain, lethargy and encephalopathy. The median time from the presentation of symptoms to PICU admission was 4.7 days (range 3-8 days).

All the children had tachypnoea and tachycardia on admission to the PICU. All patients had Pa02/Fio2 less than 300 and nine patients needed intubation and mechanical ventilation (all were intubated due to respiratory failure outside the PICU). One patient responded to oxygen supplementation via non-rebreathing mask despite decreased o2 saturation and PaO2 of 47 mmHg.

In other intubated patients, the main problem was severe hypoxia. Although they needed a low peak inspiratory pressure (PIP) for acceptable tidal volume, their oxygen saturation could not reach 85% , so peak expiratory pressure (PEEP) was increased even up to 18 and prone positioning was ordered to increase the oxygen saturation. (cases number 4 and 6). Extracorporeal membrane oxygenation (ECMO) was not available in our center.

Unfortunately, five patients died. Four of the five had chronic medical conditions. Cardiac arrhythmias occurred in three of the children who died.

Nine children had hypotension. Six children received high dose of norepinephrine(more than 0.3 micro/kilogram/minutes) in addition to other

Confidential: For Review Only

inotropes. Enoxaparin was started with the aim of antithrombotic prophylaxis in all the patients. In all patients broad spectrum antibiotics (meropenem and vancomycin) were started in addition to hydroxychloroquine (ECG was taken first and all had normal QTC; equal to or less than 0.40s) and kaletra (lopinavir/ritonavir), and intravenous immune globulin was given (IVIG) to 8 patients due to severe septic shock (except for cases number 5 and 10). In hypotensive patients, hydrocortisone, ascorbic acid, and thiamine were started.

In all of the patients, blood culture, tracheal aspirate, urine culture, and PCR for influenza A and B were negative (Table 2,3).

Patient number 3 presented with fever, abdominal pain and tachypnea. he was transferred by his parents to our center with the diagnosis of acute liver failure (Namazi hospital is the referral center for liver transplant in Iran). On arrival, he was intubated due to decreased o2 saturation and decreased level of consciousness. He had hypotension, fever and patchy ground glass infiltrations in the chest CT scan. Surprisingly, the serum bilirubin level was very high (total bilirubin was 35); the other lab data in addition to alkaline phosphatase:387 and Gamma-glutamyl transferase (GGT) was:45 are shown in Table 3.The patient tests for hepatitis A, B, C, EBV, HSV and CMV were negative and serum ceruloplasmin level and LKM Ab were in normal range; he had a negative history of taking medications or substance abuse. His parents had fever and cough and their nasopharyngeal RT-PCR for COVID 19 turned positive in both.

Table 2: clinical details of the patients

Patient number	1	2	3	4	5	6	7	8	9	10
Real-time PCR	+	+	+	+	+	+	-	-	-	-
Age(years)	4 months	3	11	13	15	16	1	1.5	3	16
Sex	male	male	male	male	male	male	male	female	female	male
Hypotension	Yes	Yes	Yes	Yes	No	Yes	Yes	Yes	Yes	Yes
Tachycardia on admission	Yes	Yes	Yes	Yes	Yes	Yes	Yes	Yes	Yes	Yes
Tachypnea on admission	Yes	Yes	Yes	Yes	Yes	Yes	Yes	Yes	Yes	Yes
Chief complaint(s)	Fever & Letharginess	Fever & cough	Fever & abdominal pain & decreased LOC	Fever & Letharginess	Fever & cough	Fever & cough	Fever & Letharginess	Fever & cough	Fever & cough	Fever & cough

Confidential: For Review Only

Previous illness	Cerebral Shunt	negative	negative	Cerebral palsy	negative	wegener granolomatosis	UPJO• with nephrostomy	negative	Immunodeficiency∞	Cerebral palsy
Convulsion	Yes	No	Yes	No	No	No	No	Yes	Yes	No
Decreased LOC*	negative	negative	positive	positive	negative	negative	negative	negative	negative	negative
Mechanical ventilation	Yes	Yes	Yes	Yes	No	Yes	Yes	Yes	Yes	Yes
ARDS# classification	Severe	moderate	Severe	Severe	Moderate	Severe	Severe	moderate	Severe	Severe
History of contact	negative	positive	positive	negative	positive	negative	negative	positive	positive	negative
Died	Yes	No	Yes	No	No	Yes	Yes	No	No	Yes

*LOC: level of consciousness

#ARDS: acute respiratory distress syndrome

• UPJO: urethropelvic junction obstruction

∞ Immunodeficiency-10 is an autosomal recessive primary immunodeficiency characterized by the onset of recurrent infections in childhood due to defective T- and NK-cell function although the severity is variable[11] .

Table 3: laboratory results on day one of admission

Confidential: For Review Only

COVID Real-time PCR	positive						negative			
Patient	1	2	3	4	5	6	7	8	9	10
Ferritin (µg/L)<br>M: 22.81-275<br>F: 4.63-204	580	1235	954	5701	750	1310	3489	1067	651	*
Typical Chest CT finding	positive	positive	positive	positive	positive	#	positive	positive	positive	positive
White blood cells(count/ml)	26100	9800	7100	11300	7500	22000	14000	12000	3800	12000
Lymphocyte	2088	1470	710	1017	1100	1100	6380	2400	760	480
Procalcitonin<br>≤ 0.3(µg/L)	0.24	30.5	10.1	1.36	0.6	*	1.8	73	0.2	*
C- reactive protein <6 (mg/L)	32	90	50	150	37	5	3	85	3	3
Creatine phosphokinase (µkat/L)<br>M:< 2.86<br>F:< 2.4	0.62	29.5	15.8	6.2	9.3	4.4	1.4	3.6	2.7	2.3
Lactate dehydrogenase (µkat/L) < 8	10.6	10.6	94.5	30.3	22.5	22.5	19.2	13.3	27.8	15.6
Troponin<br>(µg/L)< 19	32	29.2	3343	2029	1.5	450	27	68	35	1.5
D-Dimer<br>(nmol/L)<br>< 2738	10535	2957	54760	50554	7096	3993	28119	52219	7096	50554
Total bilirubin (µmol/L)5.1-17	11.9	5.1	598.6	10.2	6.8	3.4	*	5.1	10.2	*
Direct bilirubin (µmol/L)3.4-12.0	5.1	1.7	359	1.7	3.4	1.7	*	1.7	1.7	*
Aspartate transaminase (µkat/L)<br>M: < 0.62 F: < 0.52	17	0.6	33.9	0.85	0.67	1.6	*	1.2	1.1	*
Alanine aminotransferase (µkat/L) M: < 0.68<br>F: < 0.52	0.8	0.2	11.5	0.62	0.55	0.22	*	0.47	0.57	*
Creatinine (µmol/L)<br>M: 53-106<br>F: 44-97	79.5	44.2	8.8	44.2	79.5	424.3	44.2	35.3	35.3	291.7
Blood urea nitrogen (mmol/L)<br>3.6-7.1	4.6	3.9	40.3	4.6	5	18.9	3.2	1	6.7	22.1

M: male/F: female

was not performed due to his unstable condition

Confidential: For Review Only

*did not checked

Discussion

There are few studies regarding pediatric patients admitted in PICU. Pediatric data from Madrid, Spain , reported no mortality, but described one child who needed mechanical ventilation and two who needed non-invasive ventilation[12]. In a Chinese study there was only one case who needed mechanical ventilation[13]. In a cohort study in children hospital in Wuhan, three pediatric patients were admitted in the PICU, all with comorbidities and one case died[14]. In another study from Wuhan, one patient without comorbidity was admitted in PICU and survived[5].

In our study, all patients were febrile and 70% had cough, but in some studies non-critical pediatric fever was present in less than 50%[5 13 14].

In a study on 48 pediatric patients with COVID-19 admitted in 46 North American PICUs, 83% had significant pre-existing comorbidities, 73% presented with respiratory symptoms, 38% required invasive ventilation, and 23% had failure of 2 or more organ systems. The mortality rate was 4% in their study (up to the time of the report). Three patients were intubated, one patient was taking extracorporeal membrane oxygenation(ECMO), and only 25% needed vasoactive support[15].

In the beginning of the pandemic, it was assumed that the main organ involvement in COVID-19 was respiratory, but several studies have reported Kawasaki- like syndrome MIS-C later[16].

DeBiasi et al. described a 177 pediatric patients series in the Washington, DC metropolitan region; among them, 9 cases required critical care, 8 needed respiratory support, and one had Kawasaki-like shock syndrome[17].

In our study, some of our severely infected patients had elevated troponin level and fulfilling the criteria for MIS-C on arrival to PICU. Also hypotension and organ hypo perfusion, can explain the etiology of high level of troponin.

It has been shown that coronavirus infection (SARS, MERS and even; COVID-19) could damage the liver, and mild to moderate elevation of Alanine aminotransferase (ALT); decreased albumin and increased serum bilirubin levels occur frequently[18-21], but extremely high ALT and bilirubin levels in patient

Confidential: For Review Only

number 3 was noteworthy with some probable explanation like acute liver failure caused by COVID-19 or a rare complication of the disease[22].

Through reporting the characteristics of our patients, we aim to share our experience regarding COVID 19 patients.

There is a general conception that pediatric patients infected by COVID-19 have less severe symptoms and better outcomes, but severe and fatal cases occur as well.

Conflict of interest: none

Funding: The authors did not receive a specific grant for this research from any funding agency in the public, commercial or not-for-profit sectors.

Authors' Contributions: A.S. planned the study and wrote the manuscript. E.S. gathered patients' data and submitted the manuscript. A.S.D. did the data interpretation A.S.D. and M.R.K. edited the manuscript and were scientific consultant. All authors discussed the results and contributed to the final manuscript.

Acknowledgements: The authors would like to thank Shiraz University of Medical Sciences, Shiraz, Iran and also Center for Development of Clinical Research of Namazi Hospital and Dr. Nasrin Shokrpour for editorial assistance.

Confidential: For Review Only

References

1 .Lu H, Stratton CW, Tang YW. Outbreak of pneumonia of unknown etiology in Wuhan, China: The mystery and the miracle. *Journal of medical virology* 2020;92(4):401-02.

2 .Zhu N, Zhang D, Wang W. China Novel Coronavirus Investigating and Research Team. A novel coronavirus from patients with pneumonia in China, 2019 [published January 24, 2020]. *N Engl J Med*.

3 .Coronavirus N. situation report, 22. *World Health Organization. https://www. who. int/docs/default-source/coronaviruse/situation-reports/20200211-sitrep-22-ncov. pdf* 2019.

4 .Chan JF-W, Yuan S, Kok K-H, To KK-W, Chu H, Yang J, et al. A familial cluster of pneumonia associated with the 2019 novel coronavirus indicating person-to-person transmission: a study of a family cluster. *The Lancet* 2020;395(1022.23-514:(3

5 .Liu W, Zhang Q, Chen J, Xiang R, Song H, Shu S, et al. Detection of Covid-19 in children in early January 2020 in Wuhan, China. *New England Journal of Medicine* 2020;382(14):1370-71.

6 .Cao Q, Chen Y-C, Chen C-L, Chiu C-H. SARS-CoV-2 infection in children: Transmission dynamics and clinical characteristics. *Journal of the Formosan Medical Association* 2020;119(3):670.

7 .Organization WH. Multisystem inflammatory syndrome in children and adolescents with COVID-19. *Published May* 2020;15.

8 .Chu D, PaN Y, CheNg S. hui KP, KRiShNaN P, Liu Y, Ng DY, WaN CK, YaNg P, WaNg Q, PeiRiS M, PooN LLM. *Molecular diagnosis of a novel Coronavirus (2019-nCoV) causing an outbreak of pneumonia. Clin Chem* 2020;66:549-55.

9 .Zhang Z-L, Hou Y-L, Li D-T, Li F-Z. Laboratory findings of COVID-19: a systematic review and meta-analysis. *Scandinavian journal of clinical and laboratory investigation* 2020:1-7.

10 .Foust AM, Phillips GS, Chu WC, Daltro P, Das KM, Garcia-Peña P, et al. International Expert Consensus Statement on Chest Imaging in Pediatric COVID-19 Patient Management: Imaging Findings, Imaging Study Reporting and Imaging Study Recommendations. *Radiology: Cardiothoracic Imaging* 2020;2(2):e200214.

11 .Parry DA, Holmes TD, Gamper N, El-Sayed W, Hettiarachchi NT, Ahmed M, et al. A homozygous STIM1 mutation impairs store-operated calcium entry and natural killer cell effector function without clinical immunodeficiency. *Journal of Allergy and Clinical Immunology* 2016;137(3):955-57. e8.

12 .Tagarro A, Epalza C, Santos M, Sanz-Santaeufemia FJ, Otheo E, Moraleda C, et al. Screening and severity of coronavirus disease 2019 (COVID-19) in children in Madrid, Spain. *JAMA pediatrics* 2020.

13 .Lu X, Zhang L, Du H, Zhang J, Li YY, Qu J, et al. SARS-CoV-2 infection in children .*New England Journal of Medicine* 2020;382(17):1663-65.

Confidential: For Review Only

14 .Dong Y, Mo X, Hu Y, Qi X, Jiang F, Jiang Z, et al. Epidemiological characteristics of 2143 pediatric patients with 2019 coronavirus disease in China. *Pediatrics* 2020.

15 .Shekerdemian LS, Mahmood NR, Wolfe KK, Riggs BJ, Ross CE, McKiernan CA, et al. Characteristics and outcomes of children with coronavirus disease 2019 (COVID-19) infection admitted to US and Canadian pediatric intensive care units. *JAMA pediatrics* 2020.

16 .Jones VG, Mills M, Suarez D, Hogan CA, Yeh D, Segal JB, et al. COVID-19 and Kawasaki disease: novel virus and novel case. *Hospital Pediatrics* 2020;10(6):537-40.

17 .DeBiasi RL, Song X, Delaney M, Bell M, Smith K, Pershad J, et al. Severe COVID-19 in children and young adults in the Washington, DC metropolitan region. *The Journal of pediatrics* 2020.

18 .Arabi YM, Al-Omari A, Mandourah Y, Al-Hameed F, Sindi AA, Alraddadi B, et al. Critically ill patients with the Middle East respiratory syndrome: a multicenter retrospective cohort study. *Critical care medicine* 2017;45(10):1683-95.

19 .Huang C, Wang Y, Li X, Ren L, Zhao J, Hu Y, et al. Clinical features of patients infected with 2019 novel coronavirus in Wuhan, China. *The lancet* 2020;395(10223):497-506.

20 .Wu K-L, Lu S-N, Changchien C-S, Chiu K-W, Kuo C-H, Chuah S-K, et al. Sequential changes of serum aminotransferase levels in patients with severe acute respiratory syndrome. *The American journal of tropical medicine and hygiene* 2004;71(2):125-28.

21 .Xu L, Liu J, Lu M, Yang D, Zheng X. Liver injury during highly pathogenic human coronavirus infections. *Liver International* 2020;40(5):998-1004.

22 .Saeed A, Shorafa E, Shahramian I, Afshari M, Salahifard M, Parooie F. An 11-Year-Old Boy Infected with COVID-19 with Presentation of Acute Liver Failure. *Hepatitis Monthly*;20(6).

Confidential: For Review Only

Figure legends:

Figure 1: chest CT of patient number 5

Figure 2: chest CT of patient number 10

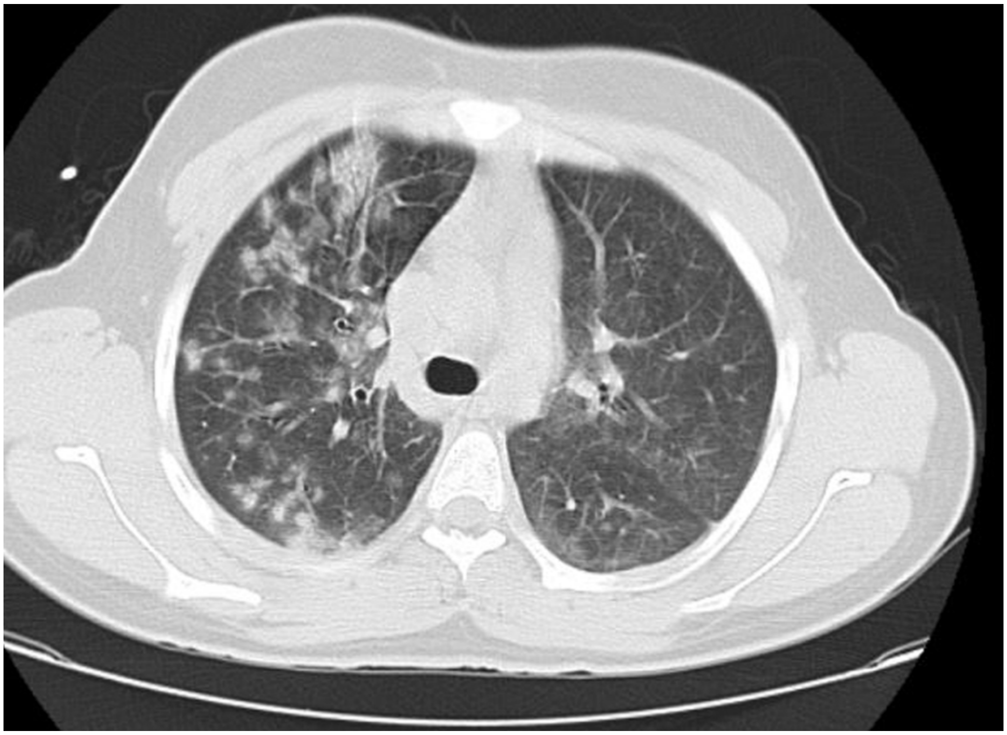

Figure 1: chest CT of patient number 5

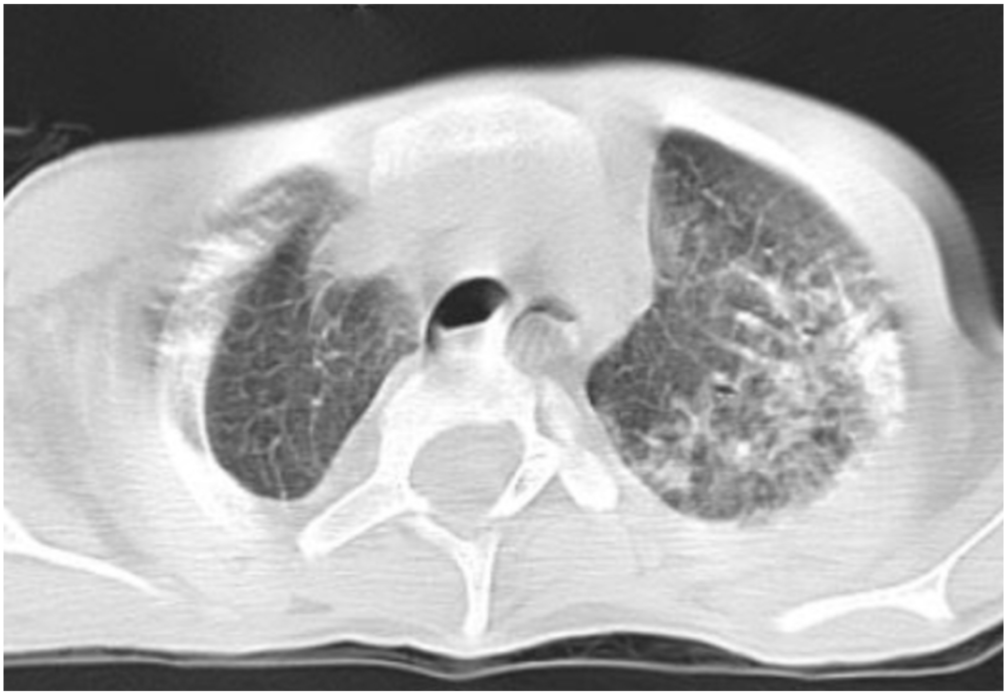

Figure 2: chest CT of patient number 10
